# Plant‐Derived Treatments for Different Types of Muscle Atrophy

**DOI:** 10.1002/ptr.8420

**Published:** 2025-01-02

**Authors:** Xingpeng Wang, Xiaofu Tang, Yunhui Wang, Shengyin Zhao, Ning Xu, Haoyu Wang, Mingjie Kuang, Shijie Han, Zhensong Jiang, Wen Zhang

**Affiliations:** ^1^ Department of Spine Surgery Shandong Provincial Hospital Affiliated to Shandong First Medical University Jinan China

**Keywords:** muscle atrophy, phytoactive compounds, phytotherapy, plant

## Abstract

With the development of medicine and chemistry, an increasing number of plant‐derived medicines have been shown to exert beneficial therapeutic on the treatment of various physical and psychological diseases. In particular, by using physical chemistry methods, we are able to examine the chemical components of plants and the effects of these substances on the human body. Muscle atrophy (MA) is characterized by decreased muscle mass and function, is caused by multiple factors and severely affects the quality of life of patients. The multifactorial and complex pathogenesis of MA hinders drug research and disease treatment. However, phytotherapy has achieved significant results in the treatment of MA. We searched PubMed and the Web of Science for articles related to plant‐derived substances and muscle atrophy. After applying exclusion and inclusion criteria, 166 and 79 articles met the inclusion criteria, respectively. A total of 173 articles were included in the study after excluding duplicates. The important role of phytoactives such as curcumin, resveratrol, and ginsenosides in the treatment of MA (e.g., maintaining a positive nitrogen balance in muscles and exerting anti‐inflammatory and antioxidant effects) has been extensively studied. Unfortunately, MA dose not have to a single cause, and each cause has its own unique mechanism of injury. This review focuses on the therapeutic mechanisms of active plant components in MA and provides insights into the personalized treatment of MA.

## Graphical abstract

Potential mechanisms of phytoactive compounds in the treatment of muscle atrophy.

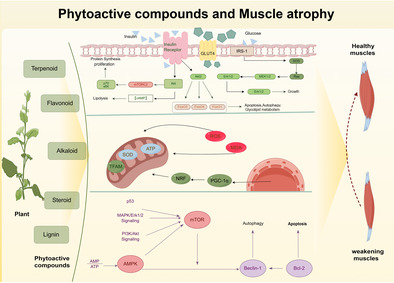

**Abbreviations**
4‐HNE: 4‐hydroxynonenal
AGEs: advanced glycation end products
Akt: protein kinase B
AMPK: adenosine 5′‐monophosphate (AMP)‐activated protein kinase
AS IV: *astragaloside IV*
ATP: adenosine triphosphate
Atrogin‐1: muscle‐specific E3 ubiquitin ligase gene‐1
C/EBP: CCAAT enhancer binding protein
CAT: catalase
CB1: cannabinoid receptor 1
CBD: cannabidiol
CHOP: C/EBP homologous protein
CL: *Codonopsis lanceolata*
Cyto C: cytochrome C
Dex: dexamethasone
DHM: *dihydromyricetin*
eIF4E: eukaryotic initiation factor 4E
Fox O: Forkhead box O
GCs: glucocorticoids
GPx: glutathione peroxidase
GR: glucocorticoid receptor
GRP‐87: glucose‐regulated protein 87
HGF: hepatocyte growth factor
IGF‐1: insulin‐like growth factor‐1
IL‐6: interleukin 6
IRS‐1: insulin receptor substrate‐1
MA: muscle atrophy
MAFbx: muscle atrophic F‐box
MDA: malonic dialdehyde
MMP: mitochondrial membrane potential
Mstn: myostatin
mTOR: mammalian target of rapamycin
mTORC1: mTOR complex 1
MuRF1: muscle RING‐finger protein‐1
NF‐κB: nuclear factor κ B
NLRP3: nucleotide‐binding oligomerization domain‐like receptor protein 3
Nrf2: nuclear factor erythroid 2‐related factor 2
PARP: caspase‐3 activation and the degradation of poly polymerase
PCS:
*Psoralea corylifolia* L. seeds
RAGE: AGEs bind to receptor for advanced glycation end products
ROS: reactive oxygen species
RosA: *rosmarinic acid*
SCs: satellite cells
SFN: *sulforaphane*
SH2: Src homology 2 domain
Sirt 1: silencing information regulator 1
SKK: *Saikokeishikankyoto*
SOCS: suppressor of cytokine signaling
SOD: superoxide dismutase
STAT3: signal transducer and activator of transcription 3
TGF‐β1: transforming growth factor β1
TLR4: Toll‐like receptor 4
TNF‐α: tumor necrosis factor α
TSC: tuberous sclerosis complex
TWEAK: tumor necrosis factor‐like weak apoptosis inducer
UPS: ubiquitin–proteasome pathway

## 1 Introduction

### 1.1 Muscle Atrophy

**FIGURE 1:**
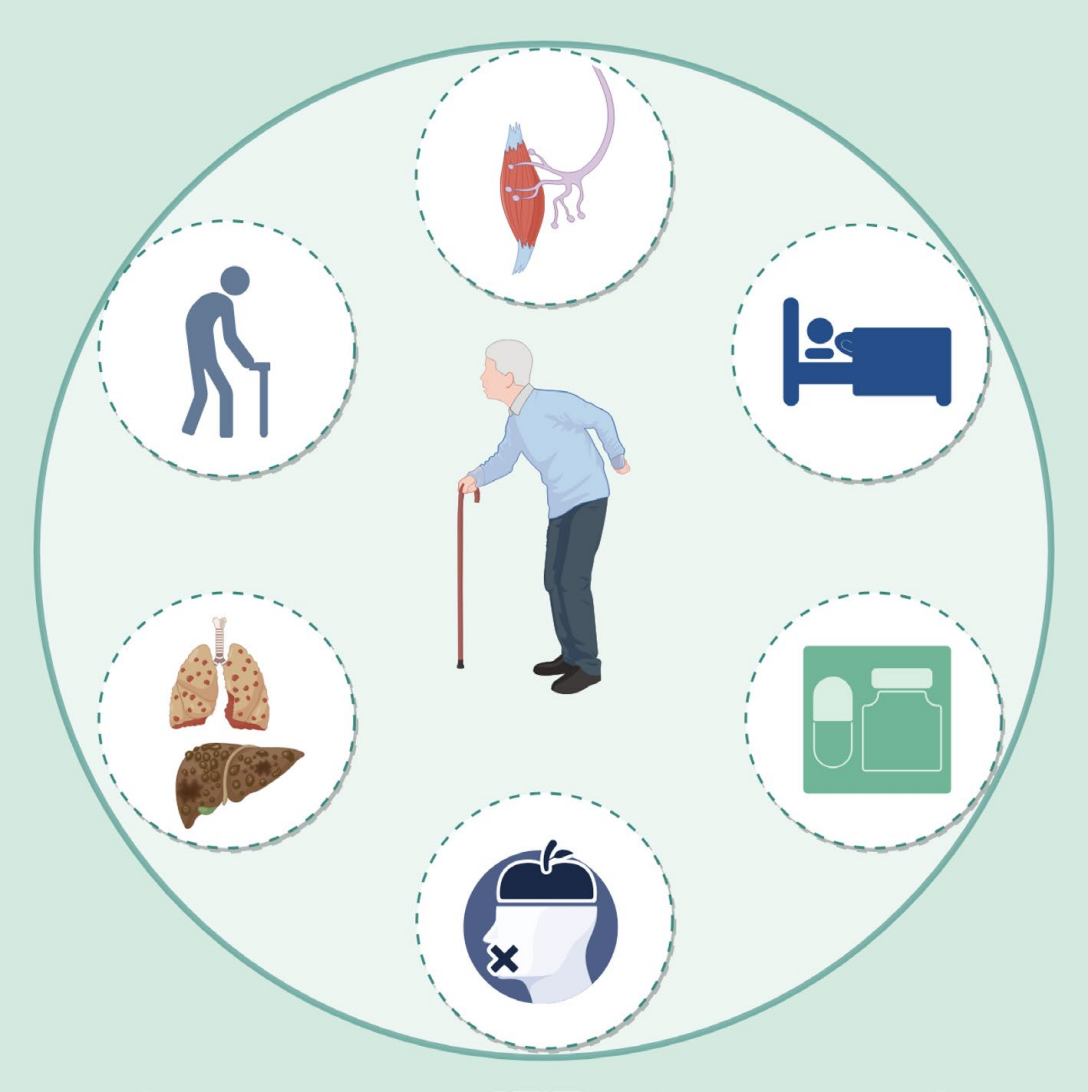
Types of muscle atrophy.

Skeletal muscle consists of multinucleated muscle fibers and represents approximately 40% of the body weight. Muscle fibers are arranged as multiple myofibrils and are divided into slow‐twitch (type I muscle fibers) and fast‐twitch (type II muscle fibers) according to characteristics such as the fiber morphology, contraction and metabolism. Fast‐twich muscle fibers can contract quickly and powerfully and are divided into type IIa myofibers (fast oxidative glycolytic fibers) and type IIb muscle fibers (fast glycolytic fibers). The function of slow‐twich muscle fibers (slow oxidative fibers) is to produce energy during aerobic exercise (Schiaffino and Reggiani 2011). The interconversion of muscle fiber types under adverse conditions eventually leads to a decrease in muscle strength (Ciciliot et al. 2013). The etiology of muscle atrophy (MA) can be divided into neurogenic atrophy (Heck and Davis 1988), disuse atrophy (Shi et al. 2023), drug‐oriented atrophy (Schakman et al. 2013), malnourished atrophy (Hsieh et al. 2020), cachexia (Bilgic et al. 2023) and age‐related sarcopenia (Cruz‐Jentoft and Sayer 2019) (Figure 1).

### 1.2 Important Factors Associated With MA

Signal transduction pathways and factors play essential roles in MA. In the insulin‐like growth factor‐1 (IGF‐1)/protein kinase B (Akt)/mammalian target of rapamycin (mTOR) pathway (Cheng et al. 2023; Han and Choung 2022; Kim, Park, and Choung 2022; Shi et al. 2023; Wang, Zhang, et al. 2023; Yeh et al. 2021, 2022), IGF‐1 promotes muscle cell proliferation through the activation of phosphorylated Akt. Akt further activates mTOR, thereby increasing enhancing protein synthesis and inhibiting protein degradation. Adenosine 5′‐monophosphate (AMP)‐activated protein kinase (AMPK) (Wang et al. 2021; Yakabe et al. 2022) is an intracellular energy metabolism sensor that is activated during energy deficiency. AMPK can not only directly phosphorylate the mTOR complex 1 (mTORC1) factor Raptor to inhibit mTORC1 but also phosphorylate tuberous sclerosis complex (TSC) to indirectly inhibit mTORC1, promote mitochondrial biosynthesis and produce an adaptive antioxidant response to combat MA. The Forkhead box O (Fox O) (Lee, Chang, et al. 2022; Liu et al. 2021; Seok et al. 2021; Yadav et al. 2021) family of transcription factors plays a significant role in regulating muscle mass and function. The interaction of Fox O with other signaling pathways, such as the Akt/mTOR and AMPK pathways, during MA is achieved through the regulation of muscle protein synthesis and breakdown. Nuclear factor κ B (NF‐κB) (Dong et al. 2022; Fang et al. 2021; Jang et al. 2021) is a transcription factor involved in the regulation of the inflammatory response and apoptosis. In MA, activation of the NF‐κB pathway causes an increase in protein degradation, leading to muscle loss. In the Ca_2+_/calpain pathway (Hou et al. 2021), increased levels of Ca_2+_ activate calpain, which triggers the degradation of muscle proteins, leading to a loss of muscle mass.

### 1.3 Phytoactive Compounds

In addition to water, sugars, proteins, fats and other necessary components in plants, components that have physiological stimulatory promotion effects on humans and various organisms are called phytoactive compounds. Phytoactive compounds including terpenoids, flavonoids, alkaloids, steroids, lignins, and minerals, have been shown to play major roles in protein anabolism, the production of mitochondria, and the regulation of inflammatory responses (Jiang et al. 2021). Although the active components of plants play vital roles in combating MA, a sufficient theoretical understanding of the effects of these drugs on the entire body is still lacking, and further research is needed.

## 2 Methods

**FIGURE 2:**
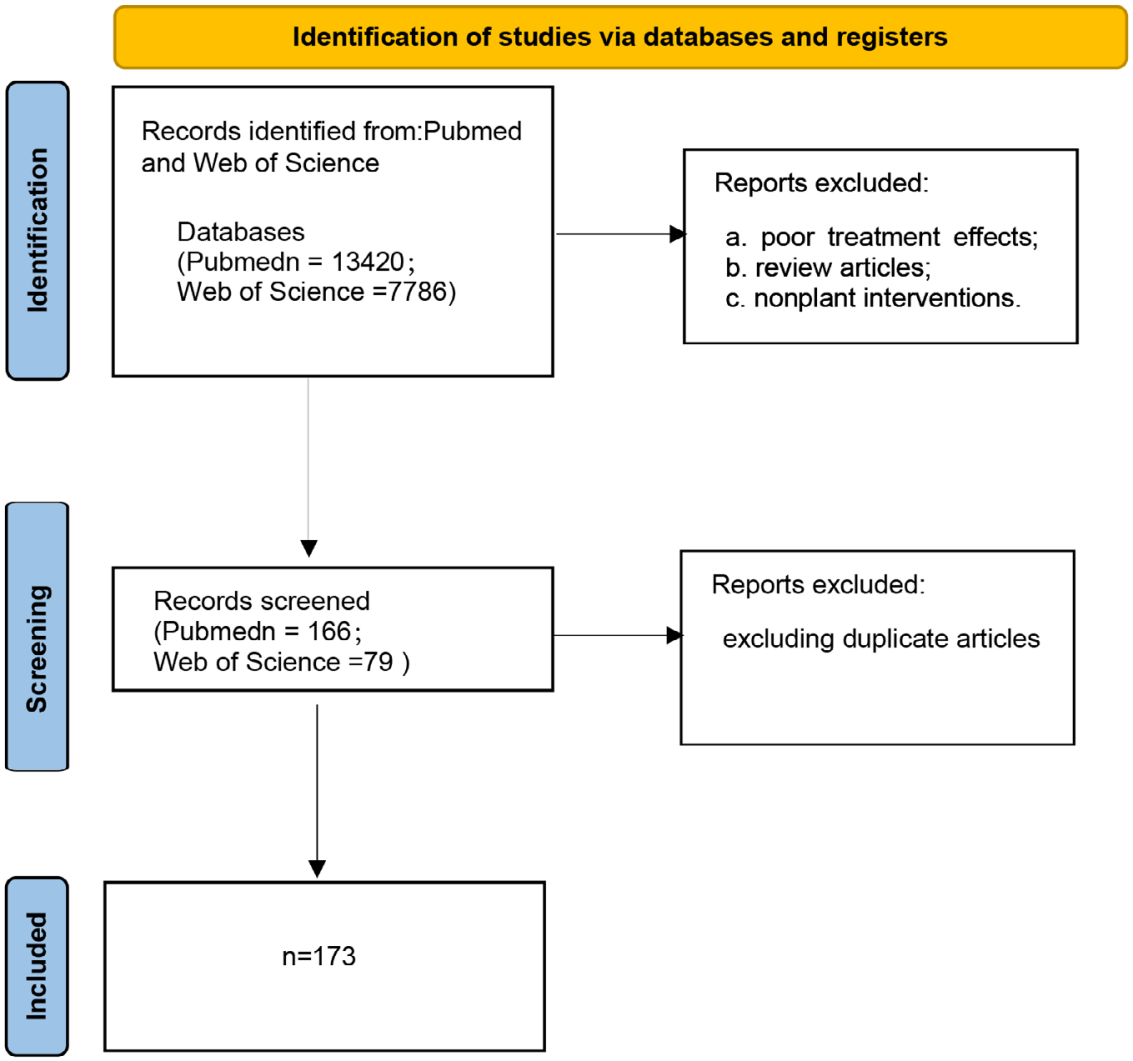
Plant‐derived substances for the treatment of muscle atrophy flowchart.

We searched PubMed and the Web of Science for the following terms: ((“muscular atrophy” [MeSH Terms] OR (“muscular” [All Fields] AND “atrophy” [All Fields]) OR “muscular atrophy” [All Fields] OR (“muscle” [All Fields] AND “atrophy” [All Fields]) OR “MA” [All Fields] OR (“sarcopenia” [MeSH Terms] OR “sarcopenia” [All Fields] OR “sarcopenia s” [All Fields])) AND “loattrfree full text” [Filter]) AND ((ffrft[Filter]) AND (2019:2023[pdat])). A total of 13,420 articles were retrieved from PubMed, and a total of 7786 articles were retrieved from the Web of Science. Both sites were searched and reviewed by two people with the following inclusion criteria: (a) muscular dystrophy model; (b) plants or plant active substances that do not constitute the main mode of intervention; and (c) effective treatment. The exclusion criteria were as follows: (a) poor treatment effects; (b) review articles; and (c) nonplant interventions. In the end, 166 and 79 articles met the inclusion criteria, and a total of 173 articles were included in the study after excluding duplicate articles (Figure 2).

## 3 Phytotherapy

### 3.1 Phytotherapy for Obesity and Diabetes‐Induced MA

The prevalence of sarcopenic obesity among adults aged 65 years and older is increasing globally. This condition, characterized by the coexistence of MA and obesity, is associated with increased rates of disability and mortality. The combination of a reduced muscle mass and excess body fat significantly impacts overall health, leading to complications that may affect mobility and quality of life in older adults (Assyov et al. 2024). The long‐term accumulation of fat not only increases the load on the body but also stresses on the joints and skeletal system and negatively affects muscle tissue. The muscles are among the main metabolic organs in the body and play a principal role in consuming energy and maintaining the basal metabolic rate. During obesity, inflammatory factors, adipocytokines, and other substances are released from adipose tissue and have negative impacts on muscles; these substances can inhibit the synthesis and repair of muscles and induce a negative nitrogen balance in muscle proteins, eventually leading to MA (Wannamethee and Atkins 2015).

According to population forecasts, the number of elderly individuals with both sarcopenia and type 2 diabetes may increase (Shou, Chen, and Xiao 2020). Diabetes is a metabolic disease characterized by a chronic increase in blood sugar levels caused by insufficient insulin secretion or insulin resistance. Sugars, lipids, and proteins are transformed through biochemical pathways such as the tricarboxylic acid cycle, and glucose homeostasis affects the balance between these factors. The PI3K/Akt pathway regulates the conversion of sugars into proteins. mTOR is the main factor that regulates downstream protein metabolism and is produced by Akt through insulin or IGF‐1 stimulation. Insulin deficiency weakens the ability of muscle cells to absorb and utilize glucose, and glucose metabolism disorders and excess free fatty acids weaken the PI3K/Akt/mTOR pathway and reduce the activity of mTORC1. In patients with diabetes, IGF‐1/PI3K/Akt signaling is decreased, Fox O phosphorylation is increased, leading to its nuclear translocation, and the transcription of muscle‐specific E3 ubiquitin ligase gene‐1/muscle atrophic F‐box (Atrogin‐1/MAFbx) and muscle RING‐finger protein‐1 (MuRF1) (Shen, Li, et al. 2022) increased levels of proinflammatory cytokines, such as tumor necrosis factor α (TNF‐α) and interleukin 6 (IL‐6), activate intracellular signaling pathways and increase the transcription of many genes encoding inflammatory mediators. For example, the transcription factor signal transducer and activator of transcription 3 (STAT3) is activated by IL‐6 and induces skeletal MA through CCAAT enhancer binding protein (C/EBP). In the case of abnormal glucose metabolism, the levels of advanced glycation end products (AGEs) are increased (Alizadeh and Kheirouri 2017); AGEs bind to receptor for advanced glycation end products (RAGE) and Toll‐like receptor 4 (TLR4), leading to the upregulation of NF‐κB and the promotion of nucleotide‐binding oligomerization domain‐like receptor protein 3 (NLRP3) inflammasome formation, which leads to cellular pyroptosis and the upregulation of MuRF‐1 and Atrogin‐1, which are involved in protein degradation and MA (Wang, Liu, et al. 2022).

**FIGURE 3:**
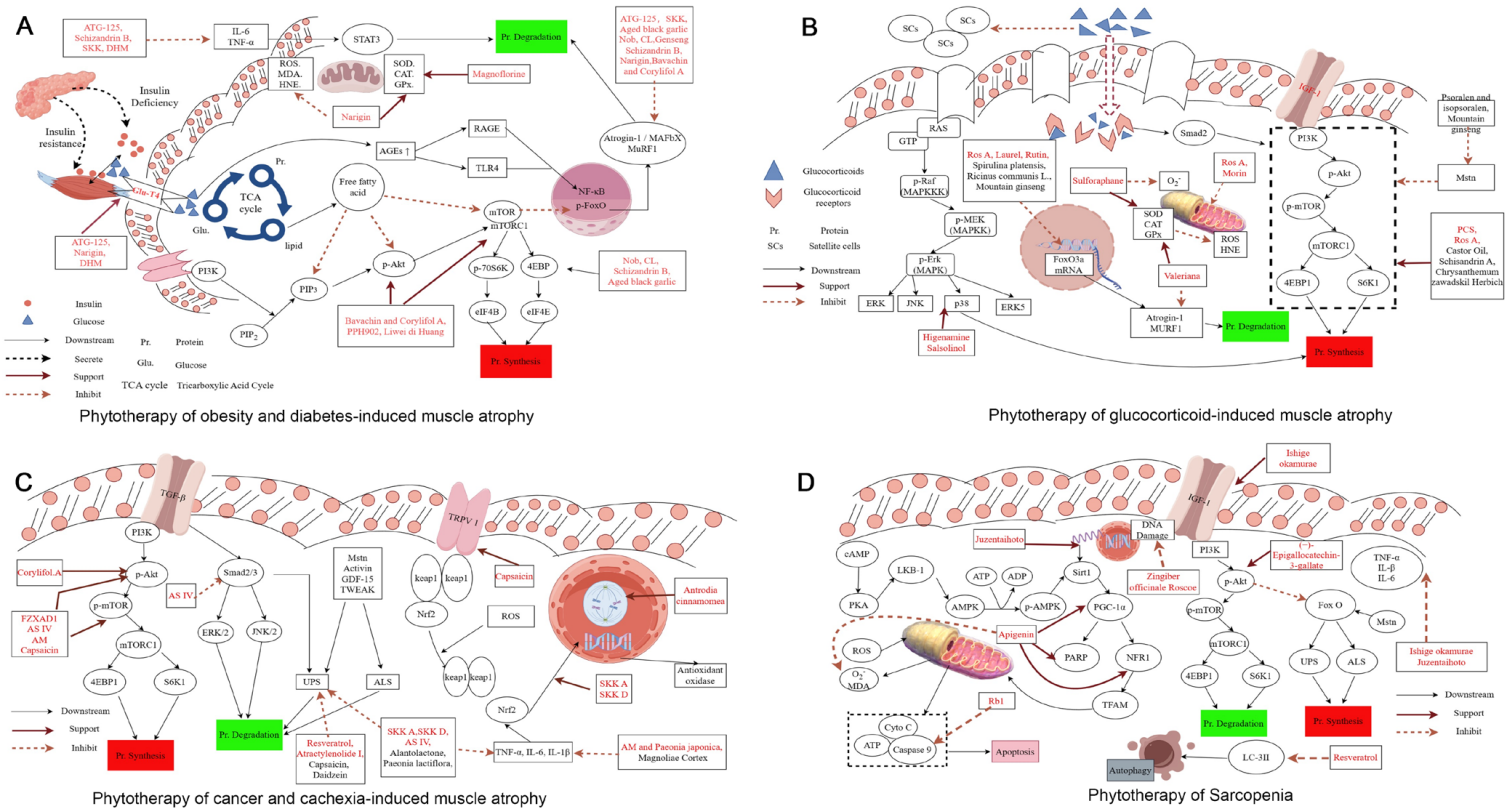
The mechanism of plant‐derived treatments for different types of MA. (A) Phytotherapy for obesity and diabetes‐induced MA. (B) Phytotherapy for glucocorticoid‐induced MA. (C) Phytotherapy for cancer and cachexia‐induced MA. (D) Phytotherapy for sarcopenia.

The imbalance of glucose and lipid metabolism is a hallmark of obesity‐ and diabetes‐induced MA. The underlying mechanism is primarily related to insulin resistance, which impairs the activation of the PI3K/Akt signaling pathway, thereby inhibiting protein synthesis via the mTOR pathway. Inhibition of the Akt signaling pathway also removes suppression on FOXO1 and FOXO3, enhancing the activity of the UPS and the autophagy‐lysosome pathway, leading to increased protein degradation. Hyperglycemia induces oxidative stress through the activation of the polyol pathway, accumulation of AGEs, and excessive ROS production. Abnormal lipid metabolism and elevated levels of free fatty acids (FFA) result in intramuscular lipid deposition. Lipid infiltration not only impairs insulin signaling but also directly damages muscle tissue through FFA‐induced oxidative stress and the secretion of adipokines, such as leptin and adiponectin. Therefore, researchers focus on restoring normal glucose and lipid metabolism in the treatment of this type of muscle atrophy, while simultaneously addressing strategies to enhance protein synthesis and mitigate inflammation and oxidative stress (Table 1 and Figure 3A).

**TABLE 1:** Phytotherapy of obesity and diabetes‐induced muscle atrophy.

Plant	Active ingredients	Mechanism	Methods of inducing muscle atrophy	PMID
	Nobiletin	1. Partial restoration of the mRNA expression of ACTA1, TMP1, troponin complexes (TNNC2, TNNT1, and TNNT3) and MYH1, MYH2 and MYH4 2. The proportions of p‐S473‐Akt/Akt and p‐Ser2448/mTOR/mTOR proteins were partially restored 3. Activates Akt and block Fox O3a/MAFbx/MuRF1 signaling to improve skeletal muscle atrophy	D‐galactose‐treated mice	37111020 (Wang, Zhang, et al. 2023)
Luteolin		1. Reduces plasma FGF21 and SPARC levels, and increases plasma LIF concentration 2. Reduces Fox O1, Fox O3, and MuRF‐1 levels 3. Inhibits the expression of Cd36, Ldlr, TNF‐α and Tlr2, and increase the expression of anti‐inflammatory factor Adipoq 4. Decreases plasma IFN‐γ and IL‐1β concentrations 5. Inhibits the activation (phosphorylation) of p38, MAPK and JNK	Obese and high‐fat diet‐fed mice	36708177 (Kim, Shin, and Kwon 2023)
Aged black garlic (*Allium sativum* L.) and aged black elephant garlic (*Allium ampeloprasum* L.)	S‐methyl‐L‐cysteine/L‐proline	1. Reduces the expression of Atrogin‐1 and MURF‐1 2. Increases the expression levels of MyHC and PGC1α and p70S6K 3. The Akt/mTOR/p70 S6K pathway enhances myogenic differentiation and myotube hypertrophy 4. Increases the mRNA expression levels of mitochondria‐related genes (including NRF 1 and TFAM)	High‐fat‐diet‐fed mice	37163777 (Chae et al. 2023)
*Psoralea corylifolia* L.	Bavachin and corylifol A	1. Reduces the expression of TNF‐α and IL‐6 2. Decreases the expression of Mstn, Atrogin‐1 and MuRF1 3. Increases the levels of p‐AKT and p‐mTOR/p‐S6K/p‐4EBP1 4. Increases the expression levels of p‐AMPKα and PGC‐1α, NRF1 and TFAM, and promotes the expression of fusion (OPA1, MFN1, and MFN2) and fission (FIS1 and DRP1) related proteins 5. Increases the activity of the PINK1/parkin pathway and upregulate muscle mitophagy to improve mitochondrial quality	Diabetes‐induced muscle atrophy	36671000 (Yeon et al. 2023)
Saikokeishikankyoto		1. Increases the activity of the SIRT1 promoter 2. Reduces the expression of inflammatory cytokines, as well as the transcription of the ubiquitin ligases Atrogin‐1 and MuRF‐1	Kkay mice	34997408 (Ou et al. 2022)
Codonopsis lanceolata	Tangshenoside I	1. Activates the PI3K/Akt/mTORC1 pathway, increases the phosphorylation of p70S6K and 4E‐BP1, increases muscle protein synthesis, and reduces muscle protein degradation by inhibiting the expression of MuRF‐1/Atrogin‐1 2. Reduces the levels of the TG synthesis‐related factors: SREBP‐1c, DGAT2 and SCD1 expression, increases the expression of the factors of CPT1, UCP3, and ACOX1 involved in fatty acid oxidation	High‐fat‐diet‐fed mice	35026519 (Han and Choung 2022)
Fruit of Schisandra chinensis	Schisandrin B	1. Inhibits the phosphorylation p38 MAPK and JNK 2. Inhibits the expression of the inflammatory factors TNFα, IL‐1β and MCP1 inflammatory factors 3. Downregulates p65 to inhibit the nuclear translocation of NF‐κ B 4. Inhibit the Mstn/SMAD/Fox O signal transduction pathway 5. P‐AKT and p‐mTOR, S6K, and 4EBP1are upregulated to promote anabolism	Obese mice	35761679 (Yoo et al. 2022)
Naringin	2,2‐Diphenyl‐1‐picrylhydrazyl, 2,20‐azino‐bis	1. Reduces lipid accumulation, reduces serum levels of damage markers, blood glucose levels and insulin resistance in rats 2. Increases the transcription of mTOR and PGC‐1α, and reduce the transcription of Atrogin‐1 and MuRF‐1 3. The levels of SOD and CAT increase significantly, which reduce the level of MDA and enhance the antioxidant capacity 4. Increases the expression of the IRS‐1 and GluT4 proteins, and increases glucose uptake and glycogen levels	High‐fat‐diet‐induced insulin resistance in obese rats	36235772 (Termkwancharoen et al. 2022)
Propolis ethanolic extract		1. Activates the Nrf2/HO‐1 signaling pathway to resist oxidative stress caused by D‐gal 2. Inhibits p38 phosphorylation and reduces p53 expression to inhibit apoptosis	D‐Gal‐induced C2C12 cell injury	37047379 (Tian et al. 2023)
Hydrolyzed guar gum		Changes the absorption of nutrients in the intestine by increasing the production of SCFAs in the intestine and reducing the number of inflammatory cells in the LPL of the small intestine or increasing the number of anti‐inflammatory cells	Diabetic mice	35334814 (Okamura et al. 2022)
	Eugenol	1. Increase the fusion index and myotube diameter. Upregulates MHC expression and downregulates atrogen mRNA and protein levels 2. Activates PGC1α, promotes mitochondrial germination, increases the ATP content and complex I enzyme activity	Diabetes‐induced muscle atrophy	36424928 (Jiang et al. 2022)
Ginseng	Gintonin‐enriched fraction	1. Promotes the activation of PGC1a, NRF1, and TFAM 2. Reduces the expression of Atrogin‐1 and MuRF1 3. Induces the expression of myogenic regulatory factors (MRFs)	High‐fat diet‐induced muscle atrophy	35600770 (Jin et al. 2022)
	Ginsenoside compound K	1. Inhibits eIF2a phosphorylation and CHOP expression to reduce endoplasmic reticulum stress 2. Increases AMPK levels and autophagy 3. Increases the phosphorylation of p38 and Akt	Obesity‐induced muscle atrophy	35600773 (Kim, Pyun, et al. 2022)
	Dihydromyricetin	1. Reduces serum energy metabolism and the levels of inflammatory factors such as IL‐1β, IL‐6, TNF‐α, and MCP1 2. Activates AMPK, inhibit NF‐κ B, and then inhibits the expression of Atrogin‐1 and MuRF‐1 3. Increases insulin sensitivity and glucose uptake, increases the phosphorylation of AMPK, inhibits the phosphorylation of JNK (insulin resistance inducible factor) and increases the expression of insulin signaling pathway gene IRS‐1, activates the of insulin pathway, increases the expression of glucose transporter GluT4, and stimulates the phosphorylation of mTOR and the expression of key genes in the protein synthesis pathway 4. Increases the concentration of Ca_2+_ in cells through ryanodine receptors activates CaMKK, and resists inflammation‐induced muscle atrophy of C2C12 cells through the CaMKK‐AMPK pathway	Rats with inflammation‐induced muscle atrophy and diabetes	34676967 (Hou et al. 2021)
Tinospora cordifolia	Magnoflorine	1. Oxidative stress: increases SOD activity, increases GPx (glutathione peroxidase) activity, reduces catalase activity, reduces lipid peroxide (MDA and HNE) levels, increases creatine kinase (CK) levels, reduces β‐glucuronidase activity 2. Reduces μ‐calpain, m‐calpain, and calpain 3 activities 3. Reduces TNF, IL‐6, Atrogin‐1, Bcl‐2, MuRF‐1 and Caspase‐3 expression 4. Reduces the levels of Atrogin‐1, MuRF‐1, Fn14, BCN1 and LC‐3B 5. Increases P‐Akt levels	STZ‐induced diabetic rats	33141056 (Yadav et al. 2021)
Schisandra		1. Reduces the mRNA expression of MuRF1 and Atrogin‐1 and inhibits protein degradation 2. Reduces the CREB‐mediated expression of KLF15 protein 3. Reduces p62/SQSTM1, LC3‐I, and LC3‐II levels, and inhibits the autophagy‐lysosomal pathway	STZ‐induced diabetic mice	34571935 (Choi, Yeon, and Jun 2021)
ATG‐125 (Artemisia argyi leaves, *Morus alba* L. leaves, *Leonurus japonicus* Houtt. leaves, *Capsicum annuum* L. leaves, Lophatherum gracile Brongn. leaves, *Curcuma longa* root and Glycyrrhiza uralensis root)	Chlorogenic acid, leonurine, rutin, isoschaftoside, isochlorogenic acid, quercetin, apigenin, glycyrrhizic acid, curcumin, and artemisetin	1. Reduces the sucrose‐induced increase in GluT4 levels and weakens the AMPK/Fox O3a/MuRF‐1 signaling pathway 2. Reduces HIF‐1α and NF‐κ B‐mediated inflammatory signaling 3. Increases the expression levels of PGC‐1α, Nrf1, and Tfam and improves mitochondrial dysfunction 4. Enhances the activity of IGF/Akt/mTOR pathway to promote protein synthesis	Sucrose‐induced gastrocnemius muscle atrophy	34913071 (Yeh et al. 2021)
	Sinapic acid (SA)	1. Inhibits creatine kinase (CK), lactate dehydrogenase (LDH), and MDA 2. Inhibits TNF‐α and IL‐6, increases SOD and CAT levels, and increases the total antioxidant capacity (T‐AOC) 3. Promotes NRF‐1 and PGC‐1α expression 4. Promotes the expression of BCL‐2 and inhibits the expression of Bax 5. Inhibits the expression of Atrogin‐1, MuRF‐1, CHOP, GRP‐87, and so forth	Diabetes‐induced muscle atrophy	35432808 (Liu Xianchu et al. 2021)
	Liuwei Dihuang	1. Improves the MMP and inhibits NADPH oxidase (Nox) activation and ROS production 2. Promotes the expression of IGF‐1R, Akt, and mTOR 3. Inhibitions of the expression of Fox O3a, Atrogin‐1, and MuRF‐1	Diabetes‐induced muscle atrophy	30668418 (Tseng et al. 2019)

GluT4 is an important protein for glucose transport in skeletal muscle that can be translocated to the cell membrane in response to insulin stimulation to exert its effects. GluT4 is overexpressed in obese patients but is reduced in diabetic patients due to inadequate insulin secretion or insulin resistance, resulting in hindered glucose transport and limited protein synthesis (Natalia et al. 2012). Yeh et al. reported that ATG‐125 could reduce the sucrose‐induced increase in GluT4 levels (Yeh et al. 2021); however, Termkwancharoen et al. and Houlianjie et al. team confirmed that naringin (Termkwancharoen et al. 2022) and dihydromyricetin (DHM) (Hou et al. 2021) could increase the amount of GluT4 in a diabetic mouse model. Similarly, eugenol can increase glucose uptake by increasing the translocation of GluT4, as confirmed by Jiang et al. (2022). Therefore, maintaining the steady state of GluT4 can improve insulin sensitivity and effectively slow MA.

IGF/PI3K/Akt is a key signaling pathway for protein synthesis, and many phytoactives have been shown to increase the activity of this pathway in the treatment of MA, such as nobiletin (Nob) (Wang, Zhang, et al. 2023), Codonopsis lanceolata (CL, tangshenoside I) (Han and Choung 2022; Kim, Park, and Choung 2022), aged black garlic (Chae et al. 2023), and schizandrin B (Yoo et al. 2022), have been shown to increase the activity of this pathway in the treatment of MA. These factors can increase the levels of p‐p70S6K and 4E‐BP, which are downstream targets of the PI3K/Akt/mTORC pathway, and activated p70S6K and 4E‐BP1 upregulate protein synthesis by activating ribosomal protein S6 and the translation initiation factor eukaryotic initiation factor 4E (eIF4E), respectively. Treatment with Nob restored the p‐S473‐Akt/Akt and p‐Ser2448‐mTOR/mTOR ratios and increased muscle protein synthesis (Wang, Zhang, et al. 2023). Bavachin and Corylifol A (Yeon et al. 2023) and extracts of Liuwei Dihuang (Tseng et al. 2019) can also promote muscle protein production through the Akt/mTOR axis.

To combat protein degradation in skeletal muscle, ATG‐125 attenuates the AMPK/Fox O3a/MuRF‐1 signalling pathway to combat protein degradation in skeletal muscle (Yeh et al. 2021), and Saikokeishikankyoto (SKK) decreases the transcription of the muscle‐specific E3 ubiquitin ligases Atrogin‐1 and MuRF‐1 and reduces the breakdown of muscle proteins (Ou et al. 2022). In addition, Nob (Wang, Zhang, et al. 2023), CL (Han and Choung 2022; Kim, Park, and Choung 2022), aged black garlic (Chae et al. 2023), schizandrin B (Yoo et al. 2022), bavachin and corylifol A (Yeon et al. 2023), fermented rice bran (Rusbana et al. 2020), naringin (Termkwancharoen et al. 2022), and ginseng (Jin et al. 2022) also reduce the levels of muscle‐specific E3 ubiquitin ligases, which can delay muscle degradation. A review of 12 randomized controlled trials compared the effects of ginseng intake on muscle damage in healthy adults without ginseng intake. The assessment includes two indicators: the creatine kinase level and subjective strength perception. The results showed that the experimental group that consumed ginseng presented significant improvements in creatine kinase levels and subjective strength perception, indicating that ginseng may help reduce exercise‐induced muscle damage, improve postexercise recovery capabilities, and support athletic training and competition (Munoz‐Castellanos et al. 2023). Schisandrae inhibits the autophagy–lysosome pathway (Choi, Yeon, and Jun 2021).

ATG‐125 (Yeh et al. 2021), SKK (Ou et al. 2022), schizandrin B (Yoo et al. 2022), and DHM (Hou et al. 2021) play important roles in reducing the levels of inflammatory factors such as HIF‐1α, TNF‐α, IL‐6, IL‐1β, MCP1, and p‐P65/P65 and inhibiting inflammation by preventing the nuclear translocation of NF‐κB. Notably, luteolin can increase the expression of the anti‐inflammatory factor Adipoq (Kim, Shin, and Kwon 2023). DHM (Hou et al. 2021) and eugenol (Jiang et al. 2022) can activate CaMKK by increasing the intracellular Ca_2+_ concentration through ryanodine receptors, which inhibits inflammation via the CaMKK‐AMPK pathway.

The body is constantly damaged by oxides such as reactive oxygen species (ROS) in the environment, which can damage membrane lipids to produce malonic dialdehyde (MDA) and human neutrophil elastase; the accumulation of MDA causes the cross‐linking and polymerization of macromolecules such as proteins and nucleic acids, further damaging the cell membrane. Moreover, antioxidant systems such as superoxide dismutase (SOD) and catalase (CAT) combat oxidative stress. Certain drugs, such as Nob, have the ability to lower ROS levels, thereby alleviating oxidative stress (Wang, Sun, et al. 2022), and a propolis ethanolic extract activate the Nrf2/HO‐1 antioxidant signaling pathway to enhance the activity of the antioxidant system (Tian et al. 2023). Some drugs, such as Nob, can reduce ROS production to reduce oxidative stress (Wang, Sun, et al. 2022), and other drugs, such as a propolis ethanolic extract, can activate the Nrf2/HO‐1 antioxidant signaling pathway to increase the activity of the antioxidant system (Tian et al. 2023). Other drugs can reduce the production of oxides and increase the activity of the antioxidant system. Naringin increases SOD and CAT levels to reduce MDA production (Termkwancharoen et al. 2022), and sinapic acid (Liu Xianchu et al. 2021) decreases the levels of C/EBP homologous protein (CHOP) and glucose‐regulated protein 87 (GRP‐87) and alleviates endoplasmic reticulum stress. Magnoflorine maintains the stability of CAT and increases SOD and glutathione peroxidase (GPx) activities (Yadav et al. 2021).

In summary, phytotherapy alleviates the systemic or local adverse effects caused by hyperglycemia in the treatment of this type of MA by stabilizing systemic blood glucose levels. The expression of GluT4 is a critical factor in maintaining glucose homeostasis. Naringin and dihydromyricetin exert specific effects on obesity‐ and diabetes‐induced MA by promoting glucose homeostasis through increased GluT4 expression, enhancing protein synthesis, and alleviating oxidative stress.

### 3.2 Phytotherapy for Glucocorticoid‐Induced MA

Glucocorticoid‐induced MA is a noninflammatory toxic myopathy and the most common form of drug‐induced myopathy. It was first described by Harvey Cushing in 1932. Glucocorticoid‐induced MA is usually caused by excess endogenous or exogenous glucocorticoids (GCs), with 50%–80% of Cushing syndrome patients showing muscle weakness (Wu, Liu, and Sun 2024). GC induces MA mainly by promoting protein degradation and inhibiting protein synthesis in muscle tissue. The inhibitory effect of GCs on muscle protein synthesis is characterized by the downregulation of muscle synthesis factor phosphorylation (Wu, Liu, and Sun 2024). mTORC1 is a key regulator of skeletal muscle mass, and mTORC1 activates the phosphorylates 4E‐BP1 and S6K1 to promote protein synthesis. GCs downregulates the phosphorylation of mTOR, prevents downstream reactions, and inhibits protein synthesis (Kim, Kim, Choi, et al. 2023). The Fox O family is a class of transcription factors involved in many biological processes that has received increasing attention for affecting longevity, metabolism and the tumor status, and the binding of GCs to the and glucocorticoid receptor (GR) upregulates the expression of the p85a subunit of PI3K, decreases PI3K kinase activity, inhibits downstream Akt phosphorylation, increases the nuclear translocation of Fox O3 and its transcriptional activity, and upregulates the mRNA expression of Atrogin‐1 and MuRF1 (Seok et al. 2021). Protein degradation is increased. GCs activates the ubiquitin–proteasome pathway (UPS) and the autophagy‐lysosomal system, which catabolize proteins. GCs can also cause cellular oxidative stress, increase the expression of Cbl‐b, and ultimately lead to the degradation of insulin receptor subunit‐1 (IRS‐1) and the inhibit of PI3K/Akt/mTOR pathway‐associated protein synthesis, thereby reducing muscle protein synthesis (Ulla et al. 2022). GCs upregulates Myostatin (Mstn), activates Smad2, dephosphorylates Akt, and inhibits the proliferation and differentiation of satellite cells (SCs). Furthermore, they induce ROS production, which causes a Ca_2+_ imbalance in mitochondria and produces mitochondria‐specific proteolytic activators to ultimately cause mitochondrial damage and affect muscle function (Schakman et al. 2013).

Long‐term or high‐dose application of GCs promotes the overexpression of GR. The receptor‐GCs complex reduces the proliferation and differentiation potential of muscle satellite cells and inhibits muscle growth and repair by promoting the secretion of Mstn. GCs also disrupts calcium homeostasis by impairing the function of sarcoplasmic reticulum calcium pumps, thereby affecting muscle contraction capacity. Impaired satellite cell differentiation potential and disrupted calcium homeostasis are characteristic features of GC‐induced MA (Table 2 and Figure 3B).

**TABLE 2:** Phytotherapy of glucocorticoid‐induced muscle atrophy.

Plant	Active ingredients	Mechanism	Methods of inducing muscle atrophy	PMID
Salvia plebeia R. Br. (SPR)	Ros A	1. Reduces the expression of Atrogin‐1, MuRF1 and Fox O3a, and increased the level of the MHC protein 2. Increases the phosphorylation of the Akt/mTOR/p70S6K axis 3. Increases Bcl‐2 levels and reduces Bax protein levels 4. Increases the LC3‐II/LC3‐I ratio and Beclin1 expression and reduces p62 expression to enhance autophagy 5. Inhibits the increase in mitochondrial ROS levels and improves the quantity and quality of mitochondria	Dex‐induced muscle atrophy in C2C12 myotubes	36768200 (Kim, Kim, Kim, et al. 2023)
*Nelumbo nucifera* leaf	Quercetin 3‐O‐galactoside (hyperoside), quercetin 3‐O‐β‐D‐glucuronide (miquelianin), and quercetin 3‐O‐glucoside (isoquercetin)	1. Downregulates of the ubiquitin‐proteasome system, autophagy pathway and GSDMD‐mediated pyroptosis pathway, which are involved in muscle degradation 2. Increases the phosphorylated ERK and P70S6K 3. Reduces the expression of MuRF‐1 and Atrogin‐1 4. Reduces the protein levels of Beclin‐1, LC3‐I and p62 5. The protein expression levels of NOD‐like receptor pyrin domain containing protein 3 (NLRP3), cleaved caspase‐1, and mature IL‐1β are reduced 6. Reduces the activation of NF‐κ B and IκB	Dex‐induced muscle atrophy in mice	36839161 (Kim, Kim, Kim, et al. 2023)
Rutin		The protein expression of FOX O3, MAFbx, and MuRF1 is reduced	Dex‐induced muscle atrophy	36978887 (Hah et al. 2023)
*Psoralea corylifolia* L. seed	Psoralen and isopsoralen	1. Increase the mRNA levels of MyHC1, MyHC2A, and MyHC2X in TA muscles 2. Reduce the expression levels of Atrogin‐1, MuRF‐1, and muscle Mstn and increase the protein levels of Myo G and MYH‐emb 3. Increases the phosphorylation of the Akt/mTOR/70S6K axis 4. Increases the levels of p‐p38 and Pak1 5. Reduces the level of 4‐HNE, increases the expression of the antioxidant enzymes superoxide dismutase 2 (SOD2), glutathione peroxidase (GPX) and catalase 6. Reduces the levels of the main transcription factors p‐I κ B and p‐NF‐κ B expressed by inflammatory molecules and reduces the levels of the MCP‐1 protein and serum TNF level	Dex‐induced muscle atrophy in mice	35728709 (Seo, Truong, and Jun 2022)
Valeriana fauriei root	Didrovaltrate	1. Increases the transcriptional activity of ERRγ and PPARδ and inhibits ROS activity 2. Reduces the expression of Atrogin‐1, Murf1, and Mstn 3. Inhibits the translocation of GR from the cytosol to the nucleus and inhibit the activation of Fox O3a	Dex‐induced muscle atrophy in mice	35069972 (Kim et al. 2022)
Laurel		1. Reduces the expression of Atrogin‐1/MAFbx and MuRF‐1 2. Inhibits the transcription of MuRF‐1, Fox O1 and Redd 1 to reduce skeletal muscle atrophy	Dex‐induced muscle atrophy in mice	35631169 (Jia et al. 2022)
	SFN	1. Inhibits apoptosis 2. Reduces catalase activity, prevents the increase in the O2‐level and prevents protein carbonylation	Dex‐induced muscle atrophy	35682634 (Micheli et al. 2022)
Spirulina platensis		1. Inhibits the expression of Atrogin‐1, MuRF‐1, and Fox O3 2. Decreases the level of the p‐akt protein and inhibits the expression of nuclear Fox O3a protein (inhibits the Akt/Fox O3a pathway), thereby inhibiting the protein expression of Atrogin‐1 and MuRF‐1	Dex‐induced muscle atrophy	35736168 (Lee, Chang, et al. 2022)
	Morin	1. It weakens Dex‐mediated rapid MyHC degradation 2. Reduces the expression of Atrogin‐1 and MuRF‐1 by downregulating the expression of KLF15 (the upstream regulator of Fox O3a) and Fox O3a (increasing phosphorylation) 3. Inhibits the Dex‐induced increases in Cbl‐b mRNA expression and MDA and APOP levels in plasma and tissue samples, reduces the expression of Nrf2 and SOD1, and inhibits the effect of reactive oxygen species	Dex‐induced muscle atrophy	35977398 (Ulla et al. 2022)
	Myricanol	1. Activates SIRT1 and inhibits Fox O3a 2. Induces autophagy and increases the clearance of degraded proteins 3. Increases PGC‐1α activity	Dex‐induced muscle atrophy	30793539 (Shen et al. 2019)
*Matricaria chamomilla*	Chlorogenic acid, luteolin‐7‐O‐glucoside, patulitrin, apigenin‐7‐O‐glucoside, herniarin, and (E)‐tonghaosu	1. Inhibits of MuRF1 transcription 2. Increases the expression of MyoD and Myogenin‐1 3. Increases the expression of TFAM	Dex‐induced muscle atrophy	34247561 (Park et al. 2021)
*Ricinus communis* L.	Castor oil plant (*Ricinus communis* L.) leaf extract	1. Increases the expression of MyoD and MyoG 2. Increases the phosphorylation level of FOX O3 and decreases the expression of Atrogin‐1 and MuRF1 3. Restores mitochondrial oxygen consumption (OCR) and reduces ROS production 4. Restores the phosphorylation levels of mTOR, Akt, S6K, and 4‐EBP1 5. Increases the activities of SOD, CAT and GSH, activates Nrf2, and reduces the production of oxidizing substances	Dex‐induced muscle atrophy	35865958 (Lee, Kim, et al. 2022)
Mountain ginseng		1. Reduces the expression of MuRF‐1, Atrogin‐1, pERK1/2, Fox O3a, Fox O1, and Mstn 2. Increases the myotube diameter and MyHC, HSP90, p‐Akt, and follistatin levels 3. Reduces the enrichment of GR, Fox O3a, and RNA polymerase II at the promoter	Dex‐treated rats	33161026 (Seok et al. 2021)
*Chrysanthemum zawadskil* Herbich		1. Increases the level of the MHC protein, decrease the expression of Atrogin‐1, MuRF‐1, and Mstn and increases the expression of Myo D and Myo G 2. Increases the phosphorylation of Akt, mTOR, and their targets S6K and 4EBP1 to promote protein synthesis 3. Reduces the protein expression of Atrogin‐1 and MuRF‐1, reduces the phosphorylation of Mstn and Smad3, and alleviates the degradation of protein 4. Increases the transcriptional activity of PPARδ and ERRγ, and inhibits mitochondrial respiratory chain damage 5. Inhibits the translocation of GR to the nucleus and weakens the upregulation of REDD1 and TLF15	Dex‐induced muscle atrophy	33485066 (Lee, Kim, Nirmala, et al. 2021)

Inhibiting protein degradation and oxidation are two aspects of treatment in people with MA who have been receiving glucocorticoids for a long period because the long‐term use of glucocorticoids increases protein degradation in muscle (Cai et al. 2024) and damages mitochondria (Shen et al. 2019). Some plant‐derived agents, such as *Psoralea corylifolia* L. seeds (PCS, Psoralen and isopsoralen) (Seo, Truong, and Jun 2022), Salvia plebeia R. Br. ([SPR], rosmarinic acid [RosA]) (Kim, Kim, Kim, et al. 2023), castor oil (*Ricinus communis* L.) (Lee, Kim, et al. 2022), schisandrin A and *Chrysanthemum zawadskil* Herbich (Lee, Kim, Nirmala, et al. 2021), have positive effects on the phosphorylation of Akt/mTOR/70S6K or the 4E‐BP1 axis. Higenamine and salsolinol can increase protein synthesis in muscle by activating the β2‐adrenergic receptor (β2AR) and PI3K/AKT signaling pathways (Kondo et al. 2022).

Mstn plays a negative role in regulating muscle growth by inhibiting the Akt‐mediated protein synthesis pathway and muscle differentiation. In studies on psoralen, isopsoralen (Seo, Truong, and Jun 2022) and mountain ginseng (Seok et al. 2021), scholars have shown that inhibiting the expression of Mstn can alleviate MA. RosA (Kim, Kim, Kim, et al. 2023), Laurel (Jia et al. 2022), Spirulina platensis (Lee, Chang, et al. 2022), *Ricinus communis* L. (Lee, Kim, et al. 2022), rutin (Hah et al. 2023), and mountain ginseng (Seok et al. 2021) can inhibit the expression of the muscle‐specific E3 ubiquitin ligases Atrogin‐1 and MuRF‐1 by inhibiting activation of the Fox O family, thereby preventing GC‐induced protein degradation in muscle. Psoralen, isopsoralen (Seo, Truong, and Jun 2022), and Chrysanthemum zawadskil Herbich (Lee, Kim, Nirmala, et al. 2021) also reduce the expression of MuRF‐1 and Atrogin‐1, but the specific mechanisms underlying these changes have not been verified.

Current research suggests that an increase in the oxidative index is a marker of GC‐induced MA in models. When phytoactive compounds are applied, they reduce intracellular oxidation levels and visibly restore muscle homeostasis. PCS increases the expression of superoxide dismutase 2 (SOD2), GPx and CAT and decreases the expression of 4‐hydroxynonenal (4‐HNE) (Seo, Truong, and Jun 2022). In addition, the roots of *Valeriana fauriei* (Kim et al. 2022) contain hesperidin, apigenin, quercetin, and kaempferol, which scavenge ROS. Sulforaphane (SFN) prevents an increase in O2‐levels and prevents protein carbonylation by stabilizing CAT activity (Micheli et al. 2022). Morin inhibits the mRNA expression of the dexamethasone (Dex)‐induced stress‐sensitive ubiquitin ligase Cbl‐b, thereby reducing damage to the IGF‐1 signaling pathway, increasing MDA and (higher protein oxide) APOP levels, and reversing the increases in Nrf2 and SOD1 levels induced by Dex (Ulla et al. 2022). These findings prove that phytoactive compounds play a role in combating oxidative stress.

Myricanol is a constituent of *Myrica cerifera* root bark. Shen et al. reported that it could improve insulin sensitivity (by activating Sirt1) and increase muscle protein synthesis. In addition, myricanol inhibits autophagy overactivation and reduces cell and tissue damage (Shen et al. 2019).

The maintenance of normal physiological functions requires the coordinated functions of many organelles, among which the stability of membrane organelles is important for the normal function of cells, and membrane organelles are easily destroyed under conditions such as inflammation and oxidative stress. Tea‐derived substances stabilize the membrane structure in cells and can be used to alleviate MA. Herbs such as PCS (Seo, Truong, and Jun 2022) and ethanol (Park et al. 2023) inhibit the production of inflammatory factors and reduce the destruction of cell membranes through inflammatory responses. RosA (Kim, Kim, Kim, et al. 2023), ethanol (Park et al. 2023), SFN and quercetin can inhibit apoptosis by increasing Bcl‐2 levels, decreasing Bax protein levels, and decreasing the activities of Caspase‐3/9. Ros A (Kim, Kim, Kim, et al. 2023) and morin (Ulla et al. 2022) partially restored the transcription of PGC1‐α, NRF1, and TFAM in myotubes, improving mitochondrial function.

At present, the treatments for this type of muscle atrophy should focus on maintaining the protein balance and resisting oxidative stress. Phytotherapy such as Chrysanthemum zawadskil Herbich inhibits protein degradation and promotes protein synthesis. *Valeriana fauriei* roots fights against cell damage caused by oxidative stress. *Psoralea corylifolia* L. and Salvia plebeia R. Br. are used as therapeutic agents for protein metabolism and oxidative stress. By studying the mechanism of MA caused by GCs, inhibiting the occurrence of GR in muscle and improving the proliferation and differentiation potential of satellite cells are aspects worth studying. Didrovaltrate and Chrysanthemum zawadskil Herbich inhibit the translocation of GR from the cytosol to the nucleus, presenting a promising therapeutic modality.

### 3.3 Phytotherapy for Cancer and Cachexia‐Induced MA

Approximately 50%–80% of cancer patients suffer from cachexia (Argilés et al. 2014). Cachexia is characterized by significant weight loss, particularly the depletion of skeletal muscle and fat tissue (Argilés et al. 2023). Muscle tissue bears the greatest burden in patients with cancer cachexia. Typically, a dynamic balance exists between the synthesis and degradation of myosin in muscle cells to maintain the stable of muscle mass and function. However, during cancer cachexia, the cachexia‐induced factors Mstn, Activin, GDF15, tumor necrosis factor‐like weak apoptosis inducer (TWEAK), and inflammatory cytokines (IFN‐γ, TNF‐α, IL‐1‐α, and IL‐1‐β) induce proteolysis by activating the UPS and the autophagy‐lysosomal system to induce MA. Transforming growth factor β1 (TGF‐β1) is an important factor in MA that prevents muscle cell growth and differentiation, and it can phosphorylate the Smad 2/3 pathway to activate muscle‐specific E3 ubiquitin ligases or the ERK1/2 and JNK1/2 signaling pathways to induce MA. Cachexia‐induced MA decreases inhibition of the Fox O family by reducing Akt activity, which increases the transcription of Murf‐1 and Atrogin‐1 after their nuclear translocation (Bilgic et al. 2023).

Phytotherapy has shown great advantages in the treatment of cancer and cachexia‐induced skeletal MA (Table 3 and Figure 3C), especially in the inhibition of muscle protein degradation and inflammation. Fuzheng Xiaoai Decoction 1 (FZXAD1) (Cheng et al. 2023), astragaloside IV (AS IV.) (Dai et al. 2023), capsaicin (Huang et al. 2022), and *Astragalus membranaceus* (AM, formononetin) (Liu et al. 2021) increased the levels of p‐Akt and p‐mTOR and inhibited the inactivation of Akt/mTORC1 signaling. Capsaicin significantly effects on energy metabolism and glucose uptake by activating multiple signaling pathways. It promotes glucose uptake in skeletal muscle cells through the activation of the AMPK and p38 MAPK pathways while enhancing AMPK activity by stimulating ROS generation (Kim et al. 2013). Additionally, capsaicin increases intracellular calcium levels via the TRPV1 receptor and upregulates PGC‐1α, promoting fatty acid oxidation, mitochondrial biogenesis, and the formation of oxidative fibers, thereby enhancing exercise endurance and preventing high‐fat diet‐induced metabolic disorders (Luo et al. 2012). These studies suggest that capsaicin has potential therapeutic effects in improving glucose metabolism, energy metabolism, and exercise performance. In a randomized controlled trial involving 600 athletes with skeletal muscle injuries, treatment with Astragalus and *Salvia miltiorrhiza* injections resulted in significant improvements in overall treatment efficacy, serum SOD levels, serum MDA contents, and plasma creatine kinase and myoglobin levels (Wei and Jinguo 2018). Interestingly, Corylifol A can increase p‐Akt levels but not p‐mTOR levels (Zhang et al. 2023). One of the causes of MA in patients with cancer and cachexia is an increase in protein degradation. Many plant‐derived factors, such as SKK A and D (Huang et al. 2023), AS IV (Dai et al. 2023), resveratrol (Wang, Yuan, et al. 2022), alantolactone (Shen, Kuang, et al. 2022), capsaicin (Huang et al. 2022), *Paeonia lactiflora* (Jang et al. 2021), and daidzein (Zhang et al. 2021), can inhibit protein degradation by reducing muscle‐specific E3 ubiquitin ligase formation in both cancer‐ and cachexia‐induced MA. SKK A and D (Huang et al. 2023), FZXAD1 (Cheng et al. 2023), AS IV (Dai et al. 2023), alantolactone (Shen, Kuang, et al. 2022), *Paeonia lactiflora* (Jang et al. 2021), AM, *Paeonia japonica* (Lee, Lee, Moon, et al. 2021), and Magnoliae cortex (Hong et al. 2021) downregulated inflammatory factors such as TNF‐α, IL‐6, and IL‐1β to alleviate inflammatory responses. A study showed that a mixture of resveratrol and other substances can significantly inhibit proinflammatory and pro‐oxidative stimuli in tendon cells, potentially helping to prevent and treat tendinopathy (Marzagalli et al. 2024).

**TABLE 3:** Phytotherapy for cancer and cachexia‐induced muscle atrophy.

Plant	Active ingredients	Mechanism	Methods of inducing muscle atrophy	PMID/DOI
Bupleurum chinense DC	Saikosaponins A and D	1. Inhibits the expression of MuRF‐1 and enhance the expression of MyoD and dystrophin 2. Downregulates TNF‐α, IL‐6, and IL‐1β, and upregulates of IL‐10 3. Activates the PI3K/AKT/Nrf2 pathway	Skeletal muscle atrophy in chronic kidney disease	37002971 (Huang et al. 2023)
Fuzheng Xiaoai Decoction 1		1. Increases P‐Akt and P‐mTOR (Akt/mTOR) levels to reduce muscle atrophy 2. The expression levels of P‐Erk1/2 and HIF‐1α are significantly increased, indicating the MAPK signaling pathway and HIF‐1 signaling pathway were also involved in the alleviation of muscle atrophy by FZXAD1	Cancer cachexia‐induced muscle atrophy	36410574 (Cheng et al. 2023)
	Cannabinoids	Improves the infiltration of CD8^+^ T cells in colorectal cancer‐associated skeletal muscle atrophy via a cannabinoid receptor 2‐mediated pathway	Cancer cachexia‐induced muscle atrophy	36871538 (Ng et al. 2023)
	Astragaloside IV	1. Reduces the expression of the inflammatory factors TNF‐α, IL‐6, and IL1‐β 2. Inhibits TGF‐β1/Smad signaling and inhibit muscle atrophy 3. Reduces the mRNA expression levels of MuRF1 and Atrogin‐1	Sepsis‐induced muscle atrophy	36586273 (Dai et al. 2023)
*Psoralea corylifolia* L	Corylifol A	1. Inhibits the of UPS system and autophagy system 2. Inhibits the activation of thousand‐and‐one amino acid kinase 1 (TAOK1) and its downstream p38‐MAPK pathway, thereby reducing the activation of Fox O3 3. Increases p‐Akt levels but not p‐mTOR levels	Cancer cachexia‐induced muscle atrophy	37439183 (Zhang et al. 2023)
	SFN	1. Increases myoblast fusion under normal conditions through Nrf2 and ERK signaling pathways to induce hypertrophy 2. Reduces the production of ROS	Cancer cachexia‐induced muscle atrophy	36534500 (Li, Trieu, et al. 2023)
Antrodia cinnamomea	Ethanol extract of Antrodia cinnamomea	1. Restores cyclin D levels 2. Decreases the expression of p53 and p21	Cisplatin‐induced muscle atrophy	DOI: 10.1155/2023/5593854 (Liang et al. 2023)
	Saikosaponin D	1. Reduces the expression of MuRF‐1 and Atrogin‐1 2. Reduces the levels of IL‐6 and IL‐1β 3. By binding to the SH2 domain of STAT3, it specifically inhibits the phosphorylation and transcriptional activity of STAT3	Cancer cachexia‐induced muscle atrophy	36447385 (Chen et al. 2022)
	Resveratrol	Through the SIRT1/Fox O1 axis, muscle atrophy is reduced	Skeletal muscle atrophy in chronic kidney disease	35606908 (Wang, Yuan, et al. 2022)
	Alantolactone	1. Inhibits the inactivation of AKT/TORC1 signal in mice, and then inhibit the overexpression of MuRF‐1 2. Inhibits the IL‐6‐induced phosphorylation of STAT3 in myotubes, thereby protecting myotubes from muscle atrophy 3. AL inhibits the phosphorylation of p65 NF‐κ B induced by TNF‐α to a certain extent	Cancer cachexia‐induced muscle atrophy	34861585 (Shen, Kuang, et al. 2022)
	Capsaicin	1. Restores the p‐Akt/Akt ratio and mTOR levels to preserve protein in skeletal muscle 2. Reduces the expression of apoptotic proteins, such as caspase3, cleaved PARP, and the Bax/Bcl‐2 ratio 3. Capsaicin can effectively regulate TRPV1 signal transduction and cause muscle hypertrophy 4. Reduces the secretion of TNF‐α, reduces the level of MDA, and regulates oxidative stress 5. Reduces the levels of MAFbx, MuRF‐1 and Mstn, and inhibits the inflammatory response 6. Improves lysosomal function and enhances autophagy	Cisplatin‐induced muscle atrophy	36401337 (Huang et al. 2022)
*Paeonia lactiflora*		Downregulation of muscle‐specific ubiquitin E3 ligase and muscle NF‐κ B signaling and cytokine levels restores the levels of MyHC and Myo D proteins in muscle, thereby restoring skeletal muscle function and quality in cisplatin‐treated mice	Cisplatin‐induced muscle atrophy	32971160 (Jang et al. 2021)
Astragalus membranaceus	Formononetin	Phosphorylation of PI3K, Akt, and Fox O3a in muscle and C2C12 myotube of rats with CKD was significantly increased, as well as the expression of myogenic proliferation and differentiation markers, myogenic differentiation factor D (Myo D) and Mysn	Skeletal muscle atrophy in chronic kidney disease	33405354 (Liu et al. 2021)
	Daidzein	By regulating the expression of Atrogin‐1 and MuRF‐1 through the GluT4/AMPK/Fox O pathway, DDP‐induced skeletal muscle atrophy is reduced	Cisplatin‐induced muscle atrophy	33876509 (Zhang et al. 2021)
Magnoliae Cortex		1. Increases the expression of M2 macrophage markers (such as MRC1, CD163, TGF‐β and Arg‐1) in skeletal muscle and decreases the expression of M1‐specific markers (including NOS2 and TNF‐α) 2. Increases the level of IGF‐1 in skeletal muscle and the numbers of M2a and M2c macrophages	Cisplatin‐induced muscle atrophy model	33804803 (Hong et al. 2021)
	Curcumin	1. Attenuates the production of $O_{2}^{-}$, increases mitochondrial ATP levels and basal OCR, and restores the enzymatic activities of MMP and electron transport chain complexes I, II, III, and IV 2. Increases the expression levels of TFAM, PGC‐1α, and NRF‐1 to promote mitochondrial germination 3. Reduces the production of MDA and increases the levels of SOD, GSH, GPx, and glutathione reductase (GR) 4. Inhibits the expression of GSK‐3β and reduces the expression of Atrogin1 and MuRF1	CKD‐induced muscle atrophy	32531667 (Wang, Yang, Zou, Zhang, et al. 2020)

In cancer‐ and cachexia‐induced MA, systemic low‐grade chronic inflammation is a prominent characteristic. Inflammation suppresses the expression of PGC‐1α, leading to impaired mitochondrial biogenesis. Pro‐inflammatory factors and metabolic dysregulation increase ROS production, damaging mitochondrial membrane potential and weakening energy supply. Proteolysis‐inducing factors secreted by tumor cells trigger lipolysis, and excessive accumulation of FFA exerts toxic effects on muscle tissue. In the cachectic state, elevated proinflammatory cytokines accelerate muscle degradation, while simultaneously activating the NF‐κB and FOXO pathways, thereby promoting UPS‐mediated protein degradation.

Phytotherapy has several unique functions in the treatment of cancer and cachexia‐induced MA. Under physiological conditions, Nrf2 maintains cellular redox homeostasis and exerts anti‐inflammatory and anticancer effects, thereby supporting cell survival (Lv et al. 2019). SKK A and D activate the PI3K/AKT/Nrf2 pathway to inhibit inflammatory factor‐mediated oxidative stress (Huang et al. 2023). SFN induces myofiber hypertrophy by increasing normal myoblast fusion through the Nrf2 and ERK signaling pathways (Li et al. 2023). STAT3 is a protein composed of 770 amino acids with 6 functionally conserved domains, one of which is the Src homology 2 domain (SH2), that recruits and activates the STAT3 molecule, which forms a homodimer by interacting with phosphorylated tyrosine residues in the related subunit (Zou et al. 2020). STAT3 becomes overactivated in most human cancers and is often associated with poor clinical outcomes. SKK D can bind to the SH2 domain of STAT3 and specifically inhibit the phosphorylation of STAT3 and its transcriptional activity, thereby alleviating MA (Chen et al. 2022).

TGF‐β is a multifunctional cytokine that belongs to the transforming growth factor superfamily, and its overexpression induces MA (Tominaga and Suzuki 2019). In a rat model of chronic kidney disease, increased phosphorylation of the transcription factor Smad2/3 activated E3 ubiquitin ligases, leading to fibrotic protein degradation and MA (Luo et al. 2016). Inhibition of the TGF‐β/SMAD signaling pathway may combat MA. AS IV decreases the levels of TGF‐β1 and p‐Smad2/3 and inhibits TGF‐β1/Smad signaling (Dai et al. 2023).

Cannabinoid receptor 2 may have an effect on colorectal cancer‐associated skeletal MA. Recent research has suggested that cannabinoid receptor 1 (CB1) can regulate muscle metabolism and lead to muscle breakdown in individuals with cachexia (Dalle, Hiroux, and Koppo 2024). Ng et al. reported that cannabinoids improve the infiltration of CD8+ T cells through cannabinoid receptor 2 (Ng et al. 2023). In a randomized controlled trial on cannabidiol (CBD), no significant effects of CBD were observed on postexercise performance, muscle damage, or inflammatory responses (Isenmann et al. 2024).

Several scholars have focused on the regulation of the cell cycle to treat cancer cachexia‐induced MA. Liang et al. demonstrated that the ethanol extract of *Antrodia cinnamomea* could restore the level of cyclin D and reduce the expression of p53 and p21 so that the cells could remain in a proliferative and active state (Liang et al. 2023).

Curcumin is a Chinese herbal medicine that is widely used to treat hypolipidaemia, inhibit coagulation, and treat choleretic conditions. In addition, it induces malignant tumor cell differentiation, induces tumor cell apoptosis and inhibits tumor growth to exert anticancer effects. In cancer and cachexia‐induced MA, curcumin not only mitigates the effects of the primary tumor but also fights MA. Studies have shown that curcumin plays an important role in mitochondrial homeostasis (Wang, Yang, Zou, Zhang, et al. 2020; Wang, Yang, Zou, Zheng, et al. 2020). For example, curcumin increases mitochondrial germination, stabilizes the mitochondrial membrane potential, and restores the activity of the electron transport chain complex. In the steady state, mitochondria reduce oxide production and provide an adequate energy supply (Wang, Yang, Zou, Zhang, et al. 2020; Wang, Yang, Zou, Zheng, et al. 2020). A meta‐analysis on the effects of curcumin supplementation on human muscle revealed that curcumin significantly alleviates skeletal muscle damage, leading to notable improvements in plasma creatine kinase levels, muscle soreness, IL‐6 levels, and range of motion (Liu, Lin, and Hu 2024).

In this type of MA, phytotherapy focuses on addressing the adverse effects of systemic chronic inflammation. Plant‐derived bioactive compounds often exert their effects systemically, and the improvement of the cachectic environment has a positive impact on muscle health. Bupleurum chinense and Astragaloside IV reduce inflammatory factors like TNF‐α, IL‐6, and IL‐1β. Alantolactone and Corylifol A inhibit NF‐κB and related signaling pathways to suppress inflammation. Curcumin and other active ingredients (e.g., Resveratrol) maintain mitochondrial membrane potential, improve ATP production, and enhance the activity of the electron transport chain.

### 3.4 Phytotherapy for Sarcopenia

Aging‐related MA is known as sarcopenia (Cruz‐Jentoft and Sayer 2019). Aging is an unavoidable physiological phenomenon, and with age, exercise restriction, and caloric restriction exacerbate the decline in muscle mass and function, further affecting patients' quality of life. Sarcopenia and other chronic diseases interact with each other and are likely to increase the physical burden on elderly people (Damluji et al. 2023). The prevalence of sarcopenia increases significantly with age. Among older adults aged 60 years and older, the prevalence of sarcopenia is approximately 5%–13%, whereas in those aged 80 years and older, this rate may increase to over 50% (Cruz‐Jentoft et al. 2019).

The occurrence of sarcopenia is influenced by a variety of factors and may include neuronal degeneration, insufficient hormone secretion (such as growth hormone and testosterone), decreased food energy utilization, and unhealthy lifestyle habits such as a prolonged sedentary lifestyle (Bauer et al. 2019). The extensive infiltration of adipose tissue can be observed in the muscle tissue of patients with sarcopenia, and excess fat deposition affects skeletal muscle by altering the hepatocyte growth factor (HGF) signaling pathway (Li, Yu, et al. 2022). During aging, the UPS and autophagy‐lysosomal system, which are associated with muscle degradation, are overactivated, and protein degradation increases. Moreover, excess Mstn signaling in ageing muscles is critical for initiating progressive amyotrophy (Bauer et al. 2019; Cho, Lee, and Song 2022).

Aging is a key factor in the development of sarcopenia. With the onset of aging, the Notch signaling pathway declines, inhibiting the proliferation of muscle satellite cells. At the same time, the Wnt signaling pathway is aberrantly activated, promoting premature differentiation of these cells and impairing muscle regeneration. The age‐related decline in hormone levels further exacerbates muscle atrophy and fat deposition. In aged muscle cells, senescence markers such as p16 and p21 are upregulated, affecting cell proliferation and differentiation. Aging leads to a deterioration of the muscle regenerative microenvironment and a decline in stem cell function.

Phytotherapy has great advantages in the treatment of sarcopenia. These compounds have fewer negative impacts on elderly people because they originate from nature. Processes from genetic regulation to vital activities can be affected by phytoactives, revealing that they have many pharmacological effects (Table 4 and Figure 3D). As a deacetylase involved in the regulation of apoptosis and senescence, inflammation inhibition, antioxidant activity and other physiological functions, silencing information regulator 1 (Sirt 1) has attracted increasing attention (Chen et al. 2020). Sirt1 is one of the key factors in glucose and lipid metabolism that stimulates mitochondrial biogenesis through the PGC‐1α/AMPK pathway (Cantó and Auwerx 2009). Therefore, this factor may be a new target for the treatment of sarcopenia. Juzentaihoto improves insulin sensitivity and promotes protein synthesis by increasing the level of the Sirt1 mRNA (Yasuyo Morita et al. 2021). However, the authors were unable to clarify which herb produced the effect. *Ishige okamurae* (diphloroethohydroxycarmalol) increases the level of IGF‐1 in muscles, which promotes protein synthesis (Hyun et al. 2022). In addition, two traditional Chinese medicines (decoctions) can reduce the expression of Atrogin‐1 and MuRF‐1 and inhibit protein hydrolysis of proteins, both of which can also reduce the expression of inflammatory factors such as TNF‐α, IL‐1β, and IL‐6 and reduce the effect of inflammation on muscles.

**TABLE 4:** Phytotherapy for sarcopenia, disuse muscle atrophy, and denervation of muscle atrophy.

Plant	Active ingredients	Mechanism	Methods of inducing muscle atrophy	PMID/DOI
Ishige okamurae	Diphloroethohydro‐xycarmalol	1. Increases the mRNA expression of IGF‐1/PI3K/Akt 2. Reduces MuRF1/Atrogin‐1/Fox O3a mRNA expression 3. Reduces the expression of inflammatory factors such as TNF‐α, IL‐1β, and IL‐6	Sarcopenia	35689860 (Hyun et al. 2022)
	Resveratrol	Short‐term resveratrol treatment reduces the expression of Vegf‐1, CD31, and Cox‐2 and attenuates the inflammatory response	Sarcopenia	35042437 (Sirago et al. 2022)
	Icariin	1. Increases the expression of the MyHC isoforms IIx/d, IIa, IIb, and I 2. Reduces the expression of the Atrogin‐1 and MuRF‐1 proteins, and increases the ratio of p‐Fox O3a/Fox O3a 3. Promotes the degradation of the Fox O3a protein through phosphorylation at Ser318/321	Orchiectomized rats	36026561 (Yang et al. 2022)
Juzentaihoto (Astragali Radix, Atractylodis Lanceae Rhizoma, Cinnamomi Cortex, Angelica Radix, Rehmanniae Radix, Ginseng Radix, Paeoniae Radix, Poria, Cnidii Rhizoma, Glycyrrhizae Radix)		1. Increases the serum level of IGF‐1 and the level of the Sirt1 mRNA in muscle 2. Reduces the serum levels of TNF‐α and IL‐6 in muscle, as well as the mRNA levels of Atrogin1 and MuRF1	Sarcopenia	33390547 (Yasuyo Morita et al. 2021)
	Ginsenoside Rb1	1. Rb1 inhibits the accumulation of ROS and protects cells from oxidative stress, thereby attenuating H_2_O_2_‐induced cytotoxicity 2. Rb1 decreases the expression of apoptosis‐related proteins caspase‐3/9 and Bax, and restores the expression of anti‐apoptotic protein Bcl‐2 3. Rb1 exerts its antioxidant effect and prevents the apoptosis of myoblasts by targeting the core regulators of nuclear factor kappa B (NF‐κ B) signaling pathway	Sarcopenia	36466088 (Dong et al. 2022)
	Apigenin	1. It increases the oxygen consumption rate (OCR) of aged mice, increases the activity of mitochondrial respiration E electron transport chain complex I, II, and IV, improves the mitochondrial membrane potential (Δψm) and increases the ATP content in ageing mice 2. The copy number of mtDNA is increased	Sarcopenia	32857105 (Wang, Yang, Zou, Zhang, et al. 2020; Wang, Yang, Zou, Zheng, et al. 2020)
*Zingiber officinale* Roscoe		Mitigate DNA damage	Sprague Dawley (SD) rats	DOI: 10.1155/2020/3823780 (Makpol et al. 2020)
*Achyranthes bidentata* Blume	Saponin/oleanolic acid saponins	1. Increases the phosphorylation levels of PI3K, Akt, and mTOR 2. Upregulates the expression of Myo D and Myogenin, and downregulates the expression of Atrogin	Immobilize mice	37028612 (Shi et al. 2023)
Kampo formula hochu‐ekki‐to (Bu‐Zhong‐Yi‐Qi‐Tang, TJ‐41) (Ginseng Radix, Astragali Radix, Atractylodis lanceae Rhizoma, Bupleuri Radix, Angelicae Radix, Cimicifugae Rhizoma, Aurantii Bobilis Pericarpium, Zingiberis Rhizoma, Zizyphi Fructus, Glycyrrhizae Radix)		1. Downregulates of the expression of Atrogin‐1 2. Induces the phosphorylation of AMPK 3. Reduces oxidative stress and inflammation in the body	Immobilize mice	36578084 (Yakabe et al. 2022)
Codonopsis lanceolata	Tangshenoside I	1. Activates the PI3K/Akt/mTORC1 pathway, upregulates the phosphorylation of p70S6K and 4E‐BP1, increase muscle protein synthesis, and reduce muscle protein degradation by inhibiting the expression of MuRF1/Atrogin‐1 2. Upregulation of mitochondrial genesis through the SIRT1/PGC‐1α pathway	Immobilize mice	35349834 (Kim, Park, and Choung 2022)
Schisandra chinensis	Gomisin G	1. Decreases the expression of Mstn, Aatrogin‐1, and MuRF1 2. Increases the expression of mTOR and 4E‐BP1 3. Mitochondrial DNA content, ATP levels, and COX activity regulate mitochondrial genesis through the sirt1 Sirt1/PGC‐1α signaling pathway	Disuse muscle atrophy	36076532 (Yeon et al. 2022)
	Astaxanthin	1. Inhibits the production of H_2_O_2_ 2. Promotes the expression of AMPKα‐1 and peroxisome proliferator‐activated receptor (PPAR)‐γ, and Ckmt2 mRNAs 3. Inhibits the increase in ROS production driven by complex III and increases the level of the MMP 4. Inhibits the expression of cytosol and caspase‐3	Immobilize mice	33530505 (Sun et al. 2021)
	Resveratrol	Attenuation of muscle proteolysis, proteolysis markers, atrophy signaling pathways and apoptosis	Immobilize mice	34572085 (Mañas‐García et al. 2021)
*Cibotium barometz*	Chlorogenic acid	1. Improves mitochondrial antioxidant activity 2. Improves the cross‐sectional area of muscle fibers and inhibit the transformation of muscle fibers 3. Reduces the excessive production of ROS in tissues	Immobilize mice	34937825 (Jihao Xing et al. 2021)
	Linoleic acid	1. Reduces the expression of SOD1, Bax, HSP70, and FOX O1 2. Inhibits the increase in the expression of the MuRF1 and Atrogin‐1/MAFbx mRNAs	Denervation	35563168 (Lee, Lee, et al. 2022)
Lemon Peel	Eriocitrin	1. Inhibits the transcription of Atrogin‐1, MuRF‐1, and Fox O1 2. Decreases lipid peroxides and the GSSG/GSH ratio	Denervation	34189920 (Takase et al. 2021)
	Maslinic acid	Induces IGF‐1 expression and inhibits the expression of Atrogin‐1, MuRF‐1, and TGF‐β	Denervation	34578826 (Yamauchi et al. 2021)
	*T*. *cordifolia*	1. Reduces the MPO content of myeloperoxide and reduces inflammatory cell infiltration 2. Improves creatine kinase (CK) activity 3. Increases catalase activity and the GPx level and reduces SOD activity 4. Reduces the levels of β‐glucuronidase and TBARS 5. Reduces the activity of calpain and the content of the E3 ligase MuRF‐1	Denervation	32114167 (Sharma et al. 2020)
	Geranylgeraniol	Inhibits the expression of Atrogin‐1 in amyotrophic muscle, but does not increase the proliferation of skeletal muscle cells	Denervation and Dex‐induced muscle atrophy in mice	32871759 (Miyawaki et al. 2020)
Buyang Huanwu Tang (BYHWT) (Rhizoma Chuanxiong, Peach seeds, Astragalus, Pheretima, Tangkuei tail, Red flower, Paeoniae)		1. Slight inflammatory reaction, enhanced acetylcholinesterase activity (CoA) 2. Modulation of proteins in the PI3K/PKB/GSK3β/fox01Fox01 signaling pathway 3. Reduces the expression of Atrogin1 and MuRF1 4. Increases the expression of Bcl‐2, decreases the expression of Bax, caspase 9 and caspase 3, and inhibits apoptosis	Denervation	32259889 (Lan Zhou et al. 2020)
*Ficus carica* L.		1. Increases the expression of PPARα 2. Inhibits of NF‐κ B activation	Denervation	33343385 (Dai et al. 2020)
	Salidroside	1. Reduces the expression of IL‐6 and alleviates mitophagy 2. Reduces the expression of Atrogin1 and MuRF1 to reduce protein degradation 3. Reduces the expression of LC3B and PINK1 and alleviates autophagy 4. Decreases p‐STAT3 and SOCS3 levels	Denervation	31293430 (Wu et al. 2019)
	(−)‐Epicatechin	Reduces FOX O1, MAFbx, and MuRF1 levels	Complete spinal cord transection in a murine model	33151476 (Gonzalez‐Ruiz et al. 2020)

In phytotherapy, reducing mitochondrial damage may be a promising direction for the treatment of sarcopenia. The mechanisms that can cause mitochondrial damage include apoptosis, endogenous ROS, and genetic damage. When cells are damaged, proapoptotic proteins in the cytoplasm induce an increase mitochondrial membrane permeability, cytochrome C (Cyto C) is released from the mitochondria into the cytoplasm, and the complex composed of adenosine triphosphate (ATP), Cyto C and caspase‐9 is called the apoptosome. The apoptosome subsequently initiates the apoptotic cascade (Fan et al. 2005). Rb1 can inhibit the activation of caspase 3/9, block oxidative stress‐induced apoptosis, and alleviate sarcopenia (Dong et al. 2022). The quantity and quality of mitochondria play decisive roles in energy production. In older patients, mitochondria age as their bodies degenerate, making the maintenance of normal physiological activities difficult. Apigenin can improve the antioxidant capacity of ageing mouse muscle to reduce MA by increasing the activity of mitochondrial respiratory electron transport chain complexes I, II and IV, improving the mitochondrial membrane potential (MMP) in ageing mice, increasing ATP production, increasing the contents of PGC‐1α, TFAM, NRF‐1, and ATP5B, and improving mitochondrial germination and function in ageing mice. Moreover, reducing mitochondrial superoxide anion (O2‐) levels reduces MDA levels and inhibits lipid peroxidation (Wang, Yang, Zou, Zhang, et al. 2020).

Although the efficacy and safety of resveratrol in the treatment of senile diseases have been proven, extensive clinical trials are still needed to confirm the stability of this drug (Gherardi et al. 2022; Griñán‐Ferré et al. 2021; Thaung Zaw, Howe, and Wong 2021; Wong et al. 2020). Short‐term resveratrol treatment reduces the expression of Vegf‐1, CD31, and Cox‐2, improves the inflammatory response, decreases the LC3‐II/LC3‐I ratio and reduces the overactivation of autophagy (Sirago et al. 2022). In a randomized controlled trial of resveratrol combined with exercise for the treatment of functional impairment in old individuals (*n* = 60, age = 71.8 ± 6.3), patients treated with a combination of exercise and resveratrol exhibited improved skeletal muscle mitochondrial function and physical function‐related measures after 12 weeks (Harper et al. 2021).

In addition to its effects at the molecular level, phytotherapy for the treatment of sarcopenia is also associated with genetic effects. (−)‐Epigallocatechin‐3‐gallate upregulates the expression of miR‐486, stimulates p‐AKT, and inhibits Fox O1‐mediated MuRF1 and Atrogin‐1 transcription. *Zingiber officinale* Roscoe mitigates DNA damage caused by genetic ageing (Makpol et al. 2020).

As the body ages, testosterone secretion gradually decreases, which is accompanied by a decrease in muscle mass (Storer et al. 2016). Testosterone supplementation has been shown to maintain muscle function and slow ageing; however, due to the specific production and secretion mechanisms of testosterone, direct testosterone supplementation, or replacement may adversely affect other hormones in the hypothalamic–pituitary axis. Phytotherapy may be a new strategy for resolving this contradiction. As a traditional Chinese medicine, icariin is widely used to treat erectile dysfunction (Long et al. 2018) and strengthen muscles and bones (Jing et al. 2018) in men. It also has an effect on sarcopenia caused by a decrease in testosterone levels, mainly by preventing muscle degradation and increasing the number of type I muscle fibers to resist sarcopenia (Yang et al. 2022). We believe that the role of icariin in sarcopenia has not yet been examined sufficiently examined and it may have additional significant effects.

In the treatment of sarcopenia, emphasis should be placed on the suppression of aging. Aging cells impair normal physiological functions, and delaying aging is a feasible approach. Current plant‐based therapies for sarcopenia primarily focus on the systemic effects of the drugs, as aging in other systems also occurs concurrently in sarcopenia patients.

### 3.5 Phytotherapy for Disuse‐Induced MA

Patients with fractures and paralysis have to stop exercising during the course of the disease, and the muscles adapt to the new environment by changing their physiological state (Mirzoev 2020). In these patients, mitochondrial function is inhibited due to the negative nitrogen balance of the muscle in the immobilized state, which induces MA and mitochondrial gene mutations in the deloaded state. The caspase‐related mitochondrial apoptosis pathway is activated during muscle mass loss. In addition, as the level of oxidative stress in muscle increases, the cell membrane and membrane‐bound organelles are damaged, and the mitochondria, endoplasmic reticulum, and other organelles become dysfunctional (Zhang, Chen, and Fan 2007). Studies have shown a rapid loss of muscle strength after prolonged periods of bedrest (de Boer et al. 2007).

Phytoactives significantly affect the treatment of disuse‐induced MA (Table 4). Compounds such as resveratrol have been proven to increase muscle synthesis, reduce degradation, and have anti‐inflammatory and antioxidant effects (Mañas‐García et al. 2021). *Achyranthes bidentata Blume* (saponin/oleanolic acid saponins) (Shi et al. 2023) and CL promote protein synthesis by increasing the phosphorylation levels of PI3K, Akt, and mTOR, and tangshenoside I upregulates the phosphorylation of p70S6K and 4E‐BP1 (Kim, Park, and Choung 2022). Kampo formula hochu‐ekki‐to (Yakabe et al. 2022), saponin (Shi et al. 2023), tangshenoside I (Kim, Park, and Choung 2022), and resveratrol (Mañas‐García et al. 2021) reduce muscle protein degradation by inhibiting the production of muscle‐specific E3 ubiquitin ligases. Astaxanthin inhibits mitochondrial respiratory complex III‐mediated ROS production and increases MMP levels to prevent oxidative stress (Sun et al. 2021), and the Kampo formula hochu‐ekki‐to and chlorogenic acid (Jihao Xing et al. 2021) also exert various antioxidant effects (Yakabe et al. 2022). Tangshenoside I (Kim, Park, and Choung 2022) and gomisin G (Yeon et al. 2022) promote mitochondrial germination by upregulating the Sirt1/PGC‐1α pathway.

The lack of mechanical stimulation is central to disuse‐induced MA. The absence of mechanical stretch inhibits the activity of the YAP/TAZ signaling pathway, weakening the synthesis and maintenance of muscle fibers. When muscles are not sufficiently stretched or contracted, the alignment and function of muscle fibers are compromised, further exacerbating muscle degeneration. A lack of mechanical stimulation also reduces microcirculatory perfusion in muscle tissue, leading to insufficient supply of oxygen and nutrients. In a hypoxic environment, the accumulation of metabolic waste products, such as lactate, damages muscle cell function, and delays the recovery process. Treatment strategies for disuse‐induced MA include increasing the intake of high‐quality protein and resistance training. Further exploration is needed to investigate how pharmacological interventions can enhance mechanical stimulation of muscle.

### 3.6 Phytotherapy for Denervation‐Induced MA

Nerves control and regulate muscle contraction by transmitting electrochemical signals and control coordination by regulating muscle tension; by controlling muscle tension and coordination, the body is able to maintain a stable posture. Nerve stimulation promotes muscle protein synthesis and metabolism to maintain the health of muscle tissue. In addition, the stimulation of nerve endings regulates blood circulation to muscles, providing sufficient nutrients and oxygen to muscles to promote growth and repair.

Nerve stimulation plays a regulatory role in muscle protein synthesis and degradation. On the one hand, when innervation is removed, the muscles do not receive normal stimulation, resulting in the inability of the muscles to contract and move normally; furthermore, due to the removal of innervation, the synthesis of muscle proteins decreases, and degradation increases, resulting in a decrease in the number and size of muscle fibers, which triggers MA (Heck and Davis 1988). On the other hand, after nerve damage, many inflammatory cells appear in the injured area, which also exacerbates muscle damage.

Motor neuron injury leads to the loss of neural innervation of muscle fibers, impairing the muscle's ability to maintain normal function and metabolic state. The lack of depolarization signals within muscle cells disrupts muscle contraction. The neuromuscular junction (NMJ) structure is damaged, and synaptic signal transmission is completely lost. The loss of neural innervation results in the absence of neurotrophic factors (such as BDNF and NGF), which are crucial for muscle survival and regeneration. Following denervation, abnormal elevation of intracellular calcium concentrations occurs, impairing the function of the sarcoplasmic reticulum calcium pumps, activating calcium‐dependent proteases (e.g., calpain), and accelerating muscle protein degradation.

Although no definitive drug is available that can directly repair nerve or spinal cord damage, some botanical drugs have shown good results in the treatment of denervation‐induced MA (Table 4). Maslinic acid (Yamauchi et al. 2021) and geranylgeraniol (Miyawaki et al. 2020) inhibit protein degradation in subjects with denervation‐induced MA by decreasing the expression of Atrogin‐1/MuRF‐1. In particular, (−)‐epicatechin attenuates the expression of Atrogin‐1/MuRF‐1 after spinal cord injury and alleviates the effects on muscles after spinal cord injury (Gonzalez‐Ruiz et al. 2020). *T*. *cordifolia* antagonizes the proteolytic system (calpain and the UPS) to prevent muscle degradation (Sharma et al. 2020). Maslinic acid can induce IGF‐1 expression and promote protein synthesis (Yamauchi et al. 2021). Muscles without innervation are often atrophied due to an insufficient blood supply, resulting in dystrophy of muscle tissue and abnormal cellular metabolism, which further leads to muscle degeneration and atrophy. Linoleic acid reduces the expression of SOD1, Bax, and HSP70 to inhibit oxidative stress (Lee, Lee, et al. 2022). However, in a study assessing the independent and combined effects of vitamin D and conjugated linoleic acid (CLA) on myofibrillar protein synthesis rates in sedentary older adults, supplementation with vitamin D and CLA failed to improve muscle protein synthesis (van Vliet et al. 2020). Eriocitrin can inhibit the production of lipid peroxides and the antioxidant effect of GSSG/GSH (Takase et al. 2021). Nerve stimulation promotes muscle activity and maintains muscle tone and elasticity. When muscles lose their innervation, they become less active due to the lack of normal stimulation and gradually become stiff and weak, which leads to degeneration and atrophy. In summary, the elimination of innervation can lead to a lack of muscle stimulation, abnormal protein metabolism, decreased blood circulation, and decreased activity, ultimately leading to MA.

Suppressor of cytokine signaling (SOCS) induces the expression of target genes in response to environmental stimuli. SOCS‐3 can be transiently expressed after stimulation with IL‐6, aggravating cell damage. Salidroside inhibits IL‐6‐induced autophagy while inhibiting SOCS‐3 expression and attenuating denervation‐induced cellular damage (Wu et al. 2019).

Lan et al. reported that Buyang Huanwu Tang, a traditional Chinese medicine decoction, can treat denervation‐induced MA by reducing the inflammatory response, increasing acetylcholinesterase activity, and preventing muscle tonic contraction through multiple mechanisms. It reduces muscle degradation by modulating proteins in the PI3K/PKB/GSK3β/Fox O1 signaling pathway. It inhibits apoptosis by increasing the expression of Bcl‐2 and decreasing the expression of Bax, caspase‐9 and caspase‐3 (Lan Zhou et al. 2020).

TNF‐α‐induced MA is characterized by increased inflammation and oxidative responses, and improving this type of MA involves reducing inflammation and oxidative stress to reduce protein degradation. Betaine (Di Credico et al. 2023), Dioscorea nipponica Makino (Park et al. 2020) and avenanthramides (Yeo et al. 2019) reduce the expression of NF‐κB and inhibit the inflammatory response. Avenanthramides and Rg3 inhibit ROS production and stabilize mitochondrial function. In addition, betaine (Di Credico et al. 2023), Dioscorea nipponica Makino (Park et al. 2020) and Rg3 (Lee et al. 2019) can also induce the differentiation of myofibers.

The core treatment for denervation‐induced MA should focus on restoring neural innervation, repairing the NMJ, and maintaining or restoring the supply of neurotrophic factors. Additionally, regulating intracellular calcium levels, improving calcium pump function, and inhibiting the activation of calcium‐dependent proteases are also important therapeutic strategies. Buyang Huanwu Tang has been shown to increase acetylcholinesterase activity and prevent muscle spastic contractions, demonstrating a unique therapeutic effect in the treatment of denervation‐induced MA.

### 3.7 Phytotherapy for Other Types of MA

When a part of the body is infected, the immune system releases inflammatory mediators, such as cytokines and chemical mediators, to initiate the inflammatory response. These inflammatory mediators trigger local vasodilation and increase permeability to transport immune cells and nutrients to injured or infected areas (Tu and Li 2023). However, excessive or chronic inflammation can adversely affect muscles. Inflammation causes the cells in muscle tissue to be damaged and release harmful substances such as lactic acid and free radicals (Manosalva et al. 2021). Chronic inflammation can interfere with the normal synthesis and degradation processes of muscles, leading to MA and weakened muscle strength. It can also lead to a loss of muscle fibers and metabolic disorders. While inflammation is part of the natural healing process, excessive or long‐term inflammation can lead to delayed or incomplete repair. Therefore, controlling the inflammatory response has become the focus of treatment for this type of MA, and phytotherapy can not only reduce the production of inflammatory factors but also promote protein synthesis (Di Credico et al. 2023; Fang et al. 2021). In addition, many plants are metabolized by the human body and do not produce metabolites that stimulate the liver and kidneys, which makes phytotherapy an advantage in the treatment of MA (Table 5).

**TABLE 5:** Phytotherapy for other types of muscle atrophy.

Plant	Active Ingredients	Mechanism	Methods of inducing muscle atrophy	PMID/DOI
	Betaine	1. Increases the expression of Myh7, Myh2 and Myh1 2. Increases the rate of protein synthesis 3. Inhibits NF‐κ B activity	TNF‐α‐induced muscle atrophy	37013268 (Di Credico et al. 2023)
Dioscorea nipponica Makino		1. Increases MyoD and MyoG expression 2. Inhibits of NF‐κ B expression 3. Reduces the expression of Atrogin‐1 and MuRF1	TNF‐α‐induced muscle atrophy	DOI: 10.1016/j.jff.2020.104109 (Park et al. 2020)
Oat	Avenanthramides	1. Reduces the levels of IL‐6 and IL‐β and inhibits the activation of NF‐κ B 2. Reduces the expression of MuRF‐1 and Atrogin‐1 3. Reduces ROS level and increase SOD level	TNF‐α‐induced muscle atrophy	30997266 (Yeo et al. 2019)
	Rg3	1. Activation of Akt promotes myogenic differentiation and multinucleated myotube formation 2. Enhances Akt/mTOR signaling and promotes myogenesis and muscle differentiation 3. Inhibits the production of ROS and restores the content of MMP and ATP content 4. Promotes the activity and expression of PGC1α and NRF1 and TFAM 5. Inhibits the UPS	TNF‐α‐induced muscle atrophy	31271820 (Lee et al. 2019)
	SFN	1. Reduces the production of IL‐1β and inhibits the production of ROS 2. Increases the expression of E‐myosin heavy chain, myosin heavy chain and myogenin 3. Reduces the expression of TLR4, NLRP3, apoptosis‐associated speck‐like protein, and Caspase‐1	Lipopolysaccharide (LPS)‐induced skeletal muscle atrophy	36398371 (Wang, Liu, et al. 2022)
Tripterygium wilfordii	Triptolide	1. Upregulates the protein synthesis signaling (IGF‐1/p‐IGF‐1R/IRS‐1/p‐Akt/pmTOR) and downregulates the protein degradation signal Atrogin‐1 2. Upregulation of MyHC, IGF‐1, p‐IGF‐1R, IRS1, and p‐Akt3, downregulates of ubiquitin‐proteasome molecules (n‐Fox O3a/Atrogin‐1/MuRF1), proteasomal activity, autophagolysosomal molecules (LC3II/LC3‐I and Bnip3), and inflammatory mediators (NF‐κ B, Cox‐2, NLRP3, IL‐1β, and TNF‐α)	LPS‐induced skeletal muscle atrophy	33788266 (Fang et al. 2021)
	Curcumin	1. Reduces the level of MDA and ROS, and increase the content of GSH, and SOD, and CAT activities 2. Reduces the expression of Atrogin‐1, MuRF1, Mstn, and SIRT1, which are genes involved in protein degradation 3. Activates protein synthesis genes PI3K‐p85 α, Akt1, A1R, and TRPV4 4. Reduces the expression of Caspase‐3 and PARP 5. Inhibits the production levels of nitrotyrosine, 4HNE, and eNOS	Chronic forced exercise in aged mice	33950680 (Lee, Chun, Kim, et al. 2021)
	SFN	Nrf2/HO‐1 are upgraded	Serum starvation‐induced muscle atrophy	32401383 (Moon, Kim, and Kim 2020)
Chin‐shin Oolong tea	Teaghrelin	1. Induces the expression of MyoG, MHC and MyoD, and promotes muscle differentiation 2. Upregulates the Akt/mTOR signaling	Starvation‐induced muscle atrophy	31706954 (Hsieh et al. 2020)

Oxidative stress is the process by which highly reactive oxidative substances (such as free radicals) produced within cells exceed the scavenging capacity of the antioxidant system, resulting in oxidative damage to cells (Dröge 2002). Oxidative stress directly damages biomolecular structures such as proteins, lipids, and nucleic acids within muscle cells, leading to the destruction and dysfunction of cell membranes. Oxidative stress induces the release of inflammatory mediators, causing an inflammatory response (Duranti 2023). During inflammation, white blood cells further release free radicals and inflammatory mediators, creating a vicious cycle that exacerbates the degree of muscle inflammation. Free radicals can directly affect cell signaling and energy metabolism during muscle contraction, leading to weakened muscle function. Foods rich in antioxidants such as vitamin C, vitamin E (Zhao et al. 2023), and β‐carotene, and polyphenols, such as fruits, vegetables, and nuts, can be consumed to help neutralize free radicals in the body and reduce the negative effects of oxidative stress on muscles. Some plants are rich in phytoactive compounds that contain a variety of antioxidants that perform their natural antioxidant functions, in addition to a variety of other anti‐MA functions (Lee, Chun, Kim, et al. 2021; Lee et al. 2019; Moon, Kim, and Kim 2020; Wang, Liu, et al. 2022; Yeo et al. 2019).

## 4 Discussion

Plants have unique advantages as natural medicines, and the phytoactive compounds in these plants are worth exploring. The intake of high‐quality protein is mostly used in clinical practice to treat patients with MA. Although this method can increase protein synthesis by accumulating raw materials, due to the long treatment cycle and the high time cost for the short‐term effect, relevant research on the treatment of MA using phytoactive compounds is needed (Vargas‐Mendoza et al. 2022). As natural medicines, phytoactive compounds have advantages in treating MA and have a greater safety profile than chemical drugs. Phytoactive compounds are typically found in the form of plant extracts or natural products, which are natural and have relatively few side effects compared with chemically synthesized drugs. Active plant substances have good biodegradability and biocompatibility and have few toxic side effects on the body. The comprehensive effect is stronger; active plant components are often multicomponent mixtures with complex pharmacodynamic mechanisms. Compared with single chemical drugs, active substances from plants have multiple comprehensive pharmacological effects; can regulate metabolism, the antioxidant capacity, and immune function through multiple pathways, and can have a positive impact on many aspects of MA (Jugran et al. 2021). Chronic inflammation is also one of the factors leading to MA. Some phytoactive substances have anti‐inflammatory effects and can reduce inflammatory responses and promote muscle repair and reconstruction by inhibiting the release of inflammatory mediators and regulating the immune system. Some active substances from plants can directly or indirectly promote muscle growth and repair. They can stimulate protein synthesis, increase the release of insulin‐like growth factors, promote muscle cell proliferation, and improve the condition of MA (Kim, Lee, and Kim 2023).

In recent years, with the continuous exploration and research on phytoactive compounds, increasing evidence has shown that the plants can be used to treat MA (Bagherniya et al. 2022). The active components in plants have a wide range of effects and can work through several mechanisms simultaneously. Nob, tangshenoside I, and schizandrin B, among others, not only maintain the positive nitrogen balance of proteins but also exert anti‐inflammatory and antioxidant effects. Rg3 can promote mitochondrial growth and maintain mitochondrial function, in addition to the previously described effects (Lee et al. 2019). SKK can reduce protein degradation and ameliorate inflammation and oxidative stress but cannot increase protein synthesis (Ou et al. 2022). Rutin can block protein degradation, regulate autophagy and attenuate mitochondrial damage (Hah et al. 2023). Like MA, each plant or active substance has its own unique mechanism of action, and further experiments and verification are needed to determine the most suitable active plant substance for each type of atrophy to exert the optimal medicinal effect.

Maintaining normal glucose and lipid metabolism is a specific approach for treating obesity and diabetes‐induced muscle atrophy. Naringin and dihydromyricetin promote glucose homeostasis by increasing GluT4 expression. Restoring the differentiation potential of satellite cells and regulating calcium homeostasis are specific strategies for treating GC‐induced muscle atrophy. Didrovaltrate and Chrysanthemum zawadskii Herbich inhibit the translocation of the GR from the cytosol to the nucleus, thereby alleviating GC‐induced damage to satellite cell function. Reducing the adverse effects of inflammation on muscle is a key therapeutic measure for cancer and cachexia‐induced muscle atrophy. Bupleurum chinense and Astragaloside IV are effective in alleviating systemic inflammation. Aging is a core factor in the development of sarcopenia, and thus, delaying aging processes can fundamentally treat sarcopenia. The lack of mechanical stimulation is central to disuse‐induced MA, and providing appropriate mechanical stimuli is a fundamental therapeutic approach. For neurogenic muscle atrophy, the focus should be on addressing nerve damage. Buyang Huanwu Tang exerts effective action on the nerves and mitigates the impact of nerve injury.

Schisandra chinensis is considered one of the most promising candidates for the treatment of muscular atrophy. Its primary active components include lignans, such as schisandrin, pinene, and other volatile oils, along with organic acids such as citric acid, malic acid, and tartaric acid. These compounds have been studied in various types of MA. Schisacaulin D and alismoxide significantly stimulate skeletal muscle cell proliferation by increasing the number of fused myotubes and increasing the expression of myosin heavy chain (MHC) (Hien et al. 2023). Schisandrin B, a major component of Schisandra chinensis fruit, inhibits palmitic acid‐induced atrophy in C2C12 cells through the regulation of the Smad‐FOXO pathway. These findings suggest that schisandrin B may partially contribute to the protective effects of Schisandra on obesity‐induced MA (Yoo et al. 2022). Following treatment with schisandrin C, the expression of insulin receptor substrate‐1 (IRS‐1), AMP‐activated protein kinase (AMPK), PI3K, Akt, and glucose transporter type 4 (GLUT‐4) is upregulated in C2C12 cells. These findings suggest that schisandrin C may contribute to the development of novel therapeutic strategies for type 2 diabetes (T2D) (Lee, Kim, Kim, et al. 2021). Yeon, Choi, and Jun demonstrated that schisandrin A reduces protein degradation and increases protein synthesis in muscle, contributing to the amelioration of dexamethasone‐induced MA (Yeon, Choi, and Jun 2020). Additionally, schisandrin A effectively mitigates H₂O₂‐induced cytotoxicity and DNA damage by inhibiting the accumulation of ROS, restoring ATP levels, and preventing the loss of the mitochondrial membrane potential and alterations in Bcl‐2 protein expression. It also blocks H₂O₂‐induced apoptosis by inhibiting caspase‐3 activation and the degradation of poly (ADP–ribose) polymerase (PARP). These studies suggest that schisandrin A may contribute to the prevention and treatment of MA caused by oxidative stress by protecting mitochondrial function and eliminating ROS (Choi 2018). Schisandra has beneficial effects on various types of MA, although its specific mechanisms of action differ. In obesity‐ and diabetes‐induced MA, Schisandra alleviates muscle loss by stabilizing glucose levels and promoting protein synthesis. In contrast, it plays a role in stabilizing mitochondrial function in the treatment of oxidative stress‐induced MA.

Ginseng has been used clinically to treat a variety of diseases; however, its therapeutic effects on MA require further exploration. Based on current studies, we believe that ginseng has significant potential for the treatment of MA. Ginseng contains a wealth of bioactive compounds, making it effective against various types of MA. In cases of MA caused by insulin resistance, stabilizing glucose metabolism appears to be the key to treatment. For example, 20(S)‐ginsenoside Rg3 (Wang et al. 2024), ginsenoside compound K (Kim, Pyun, et al. 2022), ginsenoside CK (Li, Li, et al. 2023) and ginsenoside Rb1 (Li, Kuang, et al. 2022) act through this pathway to exert their effects. In the treatment of MA induced by hormones and cancer cachexia, compounds such as ginsenoside Rg5 (Kim, Kim, Choi, et al. 2023) and gintonin (Wijaya et al. 2021) primarily exert their effects by increasing muscle protein synthesis and inhibiting protein degradation. Similarly, in MA induced by hydrogen peroxide, where oxidative stress leads to mitochondrial dysfunction, ginsenoside Rc effectively mitigates the mitochondrial damage caused by H₂O₂‐induced cytotoxicity (Kim, Park, Kim, and Lee 2023).

SFN has been extensively studied and has varying effects on the treatment of MA caused by different factors. In obesity‐induced MA, SFN prevents obesity‐related metabolic disorders by targeting the HDAC8‐PGC1α axis, thereby enhancing mitochondrial biogenesis and function (Yang et al. 2023), and it improves glucose and lipid metabolic dysregulation caused by a high‐fat diet (Luo et al. 2024). In cancer cachexia‐induced MA, SFN activates the nuclear factor erythroid 2‐related factor 2 (Nrf2) signaling pathway, reducing oxidative stress (Li, Trieu, et al. 2023). In inflammation‐induced MA, SFN also exerts anti‐inflammatory effects through the TLR4 and NLRP3 signaling pathways (Wang, Liu, et al. 2023). In a study by Komine et al. on the effects of SFN on muscle soreness and damage caused by eccentric exercise in young adults, the intake of SFN before and after exercise increased the expression of the Nrf2 target gene NQO1 and suppressed muscle soreness several days after exercise (Komine et al. 2021).

Compared with chemical drugs, phytoactive compounds are more reactive and have fewer adverse effects on the human body. The abundant active components in plants produce wider and more potent pharmacological effects that may be synergistic. However, due to the wide variety of phytoactive compounds, screening safe and effective plants has become a new challenge, and existing studies are more focused on animal models and less focused on clinical effects. This review summarizes the prior research and provides insights and ideas for the future use of phytoactive compounds in the treatment of MA.

## 5 Conclusions

Based on our research, a growing body of work has focused on plant bioactive compounds for the treatment of muscle atrophy; however, few clinical studies in this area exist. In in vitro and animal studies, different research groups have employed various muscle atrophy models, each involving distinct mechanisms leading to muscle degradation. Despite the diversity in muscle atrophy types, certain patterns can still be identified across these conditions. Although our study failed to find specific “answers” for different types of MA, we found that despite the different pathogenic factors, the pathogenic mechanisms of MA are similar, and different types of muscular atrophy have different mechanistic characteristics. Obesity and diabetes‐induced MA are closely associated with imbalances in glucose and lipid metabolism, which serve as critical factors in the development of MA. In these conditions, modulating glucose and lipid metabolism has been shown to effectively alleviate muscle atrophy. Impaired satellite cell differentiation potential and calcium homeostasis imbalance are characteristic features of GC‐induced MA. Reducing the accumulation of GC in the body helps restore satellite cell function. In cancer and cachexia‐induced muscle atrophy, systemic low‐grade chronic inflammation is a prominent factor contributing to muscle wasting, making the reduction of inflammation's detrimental effects on muscle function an important therapeutic strategy. Aging is a core factor in the development of sarcopenia, and delaying aging processes could provide a fundamental approach to treat sarcopenia. The lack of mechanical stimulation is central to disuse‐induced MA, and finding ways to restore normal mechanical stimuli to cells through pharmacological interventions is crucial for effective treatment. In neurogenic muscle atrophy, therapeutic efforts should primarily focus on addressing nerve damage. In oxidative stress‐induced muscle atrophy, mitochondrial dysfunction is a key pathogenic factor. In summary, different types of muscle atrophy (MA) share common pathogenic mechanisms but also exhibit distinct mechanisms. When studying pharmacological treatments for MA, it is essential to differentiate between the various pathogenic factors in order to identify the most effective personalized therapies.

## Author Contributions

**Xingpeng Wang:** conceptualization, data curation, investigation, writing – original draft. **Xiaofu Tang:** conceptualization, data curation, investigation, writing – original draft. **Yunhui Wang:** data curation, investigation, resources. **Shengyin Zhao:** data curation, investigation, resources. **Ning Xu:** data curation, investigation, resources. **Haoyu Wang:** data curation, investigation, resources. **Mingjie Kuang:** data curation, investigation, resources. **Shijie Han:** data curation, investigation, resources. **Wen Zhang:** conceptualization, data curation, funding acquisition, methodology, project administration, supervision, writing – review and editing. **Zhensong Jiang:** conceptualization, funding acquisition, methodology, project administration, supervision, writing – review and editing.

## Conflicts of Interest

The authors declare no conflicts of interest.

## Data Availability

The data that support the findings of this study are available from the corresponding author upon reasonable request.
